# Topology-Oblivious Random-Walk Key Relaying in Quantum Key Distribution Networks

**DOI:** 10.3390/e28060696

**Published:** 2026-06-16

**Authors:** Krišjānis Petručeņa, Sergejs Kozlovičs, Juris Vīksna, Elīna Kalniņa, Reinis Isaks, Edgars Celms, Lelde Lāce, Edgars Rencis

**Affiliations:** Institute of Mathematics and Computer Science, University of Latvia, LV-1459 Riga, Latvia; sergejs.kozlovics@lumii.lv (S.K.); juris.viksna@lumii.lv (J.V.); reinis.isaks@lumii.lv (R.I.); edgars.celms@lumii.lv (E.C.); lelde.lace@lumii.lv (L.L.); edgars.rencis@lumii.lv (E.R.)

**Keywords:** quantum key distribution, QKD networks, trusted-node relaying, random walks, topology-oblivious routing, privacy amplification

## Abstract

Quantum key distribution (QKD) networks require relaying when distant key management entities share no direct quantum link. Most relay strategies, however, rely on centralized control or globally maintained routing state. This paper asks whether useful security and efficiency can still be obtained with topology-oblivious stochastic forwarding. It studies the security-overhead trade-off in a model in which fragmented key material is relayed via random-walk variants and reconstructed under privacy amplification. The analysis asks whether strictly local forwarding can retain useful information-theoretic security (ITS). Evaluation on the GÉANT topology, representing a European academic backbone network, shows clear differences between random-walk variants. The proposed highest-score-neighbor local path-diversification heuristic reduces the probability that relayed key material passes through a compromised node. The evaluation also shows that scouting-based loop erasure significantly shortens sampled routes and improves throughput in the model. Against one- to three-node cartels, random flow protects slightly more source–target pairs than a static disjoint-multipath method on the evaluated topologies. These findings position topology-oblivious stochastic forwarding as a simpler decentralized design for QKD relaying without centralized orchestration or gossip protocols.

## 1. Introduction

Quantum key distribution (QKD) can provide symmetric keys with *information-theoretic security* (ITS) [1], relying on the principles of quantum mechanics, in particular the no-cloning theorem and the disturbance caused by measuring non-orthogonal quantum states. Since the pioneering BB84 protocol [2,3], QKD has evolved from laboratory demonstrations into metropolitan-scale and intercity deployments capable of supporting secure communication infrastructures [4,5,6]. QKD systems available on the market have limited working distance and secret key rate [7]. Typically, fiber-based QKD links operate at distances smaller than 150 km [8], with some deployments reaching 250 km [9]. To address distance limitations, deployments are organized as multi-hop QKD networks with trusted nodes [10] and a key management layer. Adjacent nodes establish pairwise secret keys using QKD links, while the key management layer is responsible for authorization, key forwarding, and key delivery. Ultimately, any two QKD network nodes—key management entities (KMEs)—can establish a shared secret key. If we believe that no node is compromised and we use one-time pad (OTP) encryption for hop-by-hop relaying, we can reclaim ITS. In practice, we must consider a threat model where some nodes are compromised and may eavesdrop on secrets. We will refer to a cooperating, malicious node set as “cartel”. In this paper, we ask whether useful *efficiency* (security-overhead trade-off) can be achieved when forwarding is topology-oblivious. If viable, this approach could simplify the decentralized key-management layer, especially in dynamic networks with nodes and edges introduced over time. By contrast, most QKD-network protocol proposals either rely on centralized or SDN-assisted orchestration [11,12], or on topology-aware path selection. In well-known distributed link-state routing protocols such as OSPF [13], routers flood link-state advertisements (LSAs), spreading local link information in a gossip-like manner so that all routers can learn the topology. Afterwards, nodes can compute end-to-end paths using the learned topology. QKD-specific OSPF-style relaying has also been proposed as a distributed alternative to centralized orchestration [14]. In this paper, we propose an alternative random-walk construction and avoid gossip protocols. We study a topology-oblivious stochastic relay scheme that we call a *random flow*. In one random flow from source *s* to destination *t*, the source splits key material into *M* fragments and emits those fragments in parallel. Each fragment is then forwarded independently using a random-walk *variant*. The destination reconstructs the recovered material and applies seeded privacy amplification to derive the final key *block* K of size θ. In this setting, exposure is the probability that one fragment from *s* to *t* traverses at least one relay in a compromised cartel. For an admissible cartel class, $\chi_{s,t}$ denotes the worst such hit probability. We also track expected hop count $h_{s,t}$ and design-threshold efficiency $\eta_{s,t}^{\tau }$, where τ is a configured upper bound on admissible-cartel exposure and $\eta_{s,t}^{\tau }$ is a model-based extracted-output proxy per consumed QKD-derived link-key bit. Our contributions are the following:

1. A topology-oblivious random-flow relaying model in which fragmented key material is forwarded by stochastic rules that avoid global adjacency knowledge, link-state maintenance, and end-to-end path computation.
2. A highest-score path-diversification heuristic that improves worst-case exposure without requiring global topology knowledge or even the node count.
3. A scouting-based loop-erasure mechanism that shortens realized payload routes, reduces queueing pressure, and eliminates self-induced cyclic waiting in the model.
4. A simulation study on reconstructed and synthetic topologies that compares the random-walk variants under exposure, hop-count, efficiency, and throughput proxies.

We parameterize the strongest possible topology-independent target by the pair connectivity κ(s,t). A cartel of size κ(s,t) positioned on a minimum *s*–*t* vertex cut can eavesdrop all transmitted key fragments, so the largest universal cartel-size target is |C|≤κ(s,t)−1. For random flow, the resulting ITS claim is conditional on the measured or assumed exposure of the chosen admissible cartel class being at most the selected threshold τ. In the evaluation, we use τ=98% as the default design threshold, report single-relay exposure for all random-walk variants, and separately give GÉANT sensitivity measurements for non-cut two- and three-node cartels. Those cartel measurements are descriptive; they do not turn the single-relay results into a blanket multi-relay guarantee.

### Scope and Limitations

The study stays at the trusted-node key-management layer and does not model physical-layer calibration, synchronization, or alternative physical-layer relaying protocols. GÉANT and NSFNET are structural proxies without inserted intermediate relays for long fiber spans. The throughput model uses a uniform 1kbit/s secret-key rate and omits classical control overhead. These choices support comparison of local forwarding heuristics, not a deployment-ready Q-KMS product. The remainder of the paper is organized as follows. Section 2 reviews the QKD-network setting and related work. Section 3 introduces the random-flow model, random-walk variants, privacy-amplification view, and loop-erasure mechanism. Section 4 presents the simulation study and comparative results. Section 5 concludes the paper.

## 2. Background and Related Work

For QKD network modeling, QKD devices are commonly abstracted as black boxes that provide adjacent nodes with a stream of QKD-link-derived symmetric keys. If one endpoint obtains such a link key, the corresponding endpoint is assumed to have established the same key, and the key is secure according to the underlying QKD protocol, up to its stated security parameters. The BB84 protocol [2,3] is the classic paradigm for quantum key distribution (QKD). Here, classical bits are represented as quantum states, e.g., polarized photons, transmitted over an optical channel. After a process of sifting and reconciliation, the communicating parties can detect the presence of any eavesdropper. One of the principal challenges in terrestrial QKD deployment is the rapid decrease in secret-key rate with increased transmission distance, primarily due to fiber attenuation and other implementation losses. As a result, except in certain experimental satellite-based scenarios [15], practical direct-transmission QKD links are generally restricted to distances of about 150 km [8]. This reflects exponential fiber loss and repeaterless rate–loss bounds for optical channels [16]. Twin-field QKD can exceed the conventional direct-transmission scaling by using single-photon interference at an intermediate measurement node, but current systems remain experimental prototypes [17,18].

### 2.1. Trusted-Node Relaying

Because of this range limit, larger deployments use relay nodes that pass key material from one hop to the next until it reaches the destination. Taken together, the QKD links and relay-capable nodes form a graph-like QKD network. The tasks of relaying, buffering, and authorizing access to key material are handled by the key-management layer. At that transport layer [19], two nodes A,B are adjacent when they share a direct QKD link. Such neighbors continuously obtain a common sequence of link keys $\left\{l_{A,B}\right\}$, usually identified by UUIDs. These keys may be buffered and then consumed for OTP encryption of data sent over an authenticated classical channel. To establish an end-to-end key between non-adjacent nodes *s* and *t*, the source can generate a fresh final key $K_{f}$, choose a relay path, and send $K_{f}⊕l$ to its first-hop neighbor. Each relay on the path decrypts, recovers $K_{f}$, and re-encrypts it for the next hop. This explains why the security model of trusted-node QKD networks depends critically on intermediate relays remaining uncompromised [10].

### 2.2. ETSI 014 and Q-KMS

**Figure 1:**
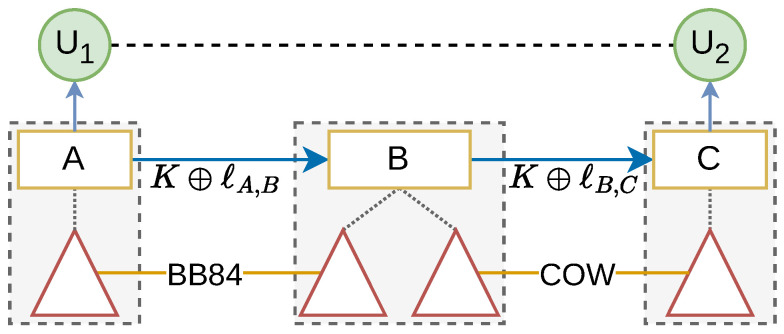
Illustration of ETSI 014-style key delivery. User $U_{1}$ requests a fresh key from KME *A* and passes the returned key identifier to user $U_{2}$, which later retrieves the same key from KME *C*. Letters *A*–*C* label the three KMEs/trusted nodes in the example relay chain. The corresponding KMEs are embedded in trusted nodes with QKD link endpoints, while the underlying Q-KMS transfers the final key $K_{f}$ hop-by-hop using OTP protection with $l_{A,B}$ and $l_{B,C}$.

The software stack that performs relaying, buffering, and authorization is often referred to as a Quantum KMS (Q-KMS). This differs from a conventional KMS, whose primary role is usually key lifecycle management: key generation, access control, auditing, and cryptographic service exposure, frequently via hardware security modules. In practice, vendors such as ID Quantique, Toshiba, and LuxQuanta typically ship proprietary Q-KMS software stacks alongside their hardware, which can limit interoperability across different vendors. At the application interface, ETSI GS QKD 014 [20] defines a RESTful HTTPS API through which applications obtain QKD-derived keys as JSON containers containing pairs of (UUID,key). The standard separates Secure Application Entities (SAEs) from Key Management Entities (KMEs), with the KMEs placed inside Trusted Nodes together with one or more QKD Entities (QKDEs) that terminate the physical QKD links. Mutual certificate-based authentication between SAE and KME is part of the model, but inter-KME relay protocols are left unspecified. Figure 1 sketches this separation between the standardized application-facing API and the non-standard relay logic behind it.

This interface should be understood as a source of key material, not as a complete substitute for end-to-end application or network security. A Secure Application Entity should not rely solely on the QKD network layer: for defense in depth, the communicating applications should still run a fresh post-quantum or hybrid key exchange in the protecting protocol, for example in a TLS 1.3 handshake or IPsec/IKEv2 exchange, and combine that result with QKD-derived material when available [21,22,23].

### 2.3. Multipath Routing

**Figure 2:**
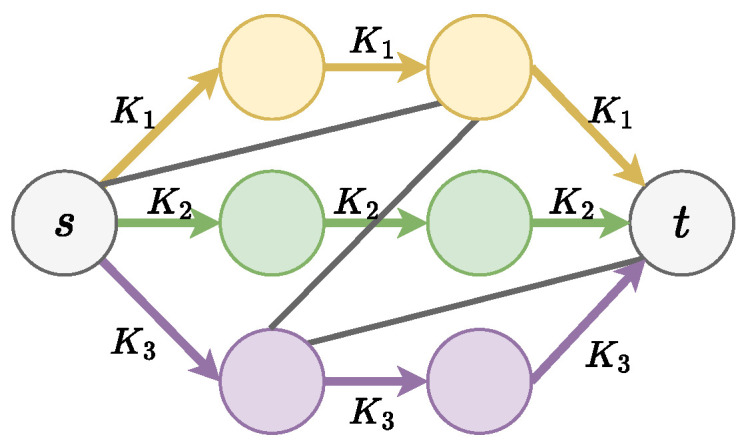
Three internally node-disjoint *s*–*t* paths (colored) with κ(s,t)=3. The colored curves distinguish the three separate paths taken by $K_{1}$, $K_{2}$, and $K_{3}$. A cartel of size 2 cannot eavesdrop on all key shares, but a cartel of size 3 on a minimum vertex cut can.

To reduce dependence on any single intermediate “trusted” node, the end-to-end key $K_{f}$ may be decomposed and sent over several strictly or partially node-disjoint routes. This family of approaches is usually called multi-path QKD. In the simplest case, the destination reconstructs $K_{f}$ from independently delivered pieces, for example by combining $K_{1},\ldots ,K_{n}$ as $K_{f}\leftarrow K_{1}⊕\cdots ⊕K_{n}$. The adversary then has to compromise at least one relay on every relevant path to recover the final key. More general (t,n) secret-sharing constructions offer tolerance against partial share leakage, albeit with additional overhead [24,25]. Such techniques are a standard way to reason about partially trusted QKD networks. Among topology-aware alternatives, deterministic routing over multiple node-disjoint paths is the main benchmark for our stochastic relaying model [26]. Figure 2 illustrates the connectivity condition. For endpoints *s* and *t* with κ(s,t)=3, the source can XOR-split $K_{f}$ across three internally node-disjoint routes. The adversary must then compromise at least one relay on every path to recover $K_{f}$. Redundant parallel links between *s* and *t* do not by themselves increase the number of node-disjoint routes. A cartel of size m=2 cannot meet all three routes, whereas a cartel of size m=κ(s,t) placed on a minimum *s*–*t* vertex cut intercepts every one of them.

For a given pair (s,t), minimum-total-length node-disjoint path sets are computable in polynomial time by Suurballe’s algorithm and Bhandari’s node-disjoint variant [27,28], and we adopt that construction as the multipath baseline in Section 4.7. Most routing proposals for trusted-relay QKD networks are topology-aware and assume some form of global or near-global state, often maintained through link-state-style control exchange and constrained by consumable key material, trust exposure, dynamic link availability, and multipath overhead [12,29,30]. Recent work has also proposed an explicitly OSPF-based distributed key-relay architecture for QKD networks, directly contrasting with our aim to avoid a link-state control plane [14]. Recent pre-relaying and decentralized key-pool work studies a different resource-management direction: QKD key material is buffered, non-reusable, and scarce, so proactive virtual key pools can reduce delay or improve allocation success at the cost of storage and relaying overhead [31,32]. Partially trusted approaches instead split key material across multiple paths or shares [24,25,26,33], while stochastic routing has also been explored before [34,35]. Recent zero-trust proposals pursue a stronger goal than the one studied here [36].

### 2.4. MDI-QKD

Measurement-device-independent QKD (MDI-QKD) offers a contrasting physical-layer design, building on side-channel-free entanglement-swapping ideas [37] and the practical protocol of Lo, Curty, and Qi [38]. In a star-style architecture, end users operate low-cost transmitters while a shared relay performs Bell-state measurements on arriving pulses [38]. The measurement relay may be untrusted: detector-side-channel attacks, including blinding and click tailoring, do not break confidentiality under the protocol model [38]. This removes trust in the detection hardware at the hub, but not in sources, channels, or classical post-processing. The protocol was first demonstrated experimentally in 2013 [39]. Compared with OTP relaying over trusted nodes, pure MDI-QKD trades a different security profile for demanding synchronization and generally lower key rates and network capacity [40,41]. Neither MDI nor trusted relaying alone achieves arbitrary long-distance scalability with current technology. Recent network studies therefore treat MDI as trusted-node *reduction* rather than elimination, and propose hybrid trusted/untrusted deployments to balance security with key-provisioning reach [42]. Operational models are converging on partially trusted relay architectures. In such networks, MDI or otherwise untrusted segments coexist with conventional trusted OTP relays, and routing selects among them according to distance, key-pool state, and residual trust [33,43]. Attacks can also exploit imperfect relay security at the routing layer even when individual QKD links appear sound [44]. The present work stays at the key-management layer for trusted-node OTP relaying and studies semi-trusted resilience via multipath diversification and topology-oblivious forwarding; it is complementary to, not subsumed by, MDI-QKD access designs.

## 3. Random Flow

At a high level, we replace deterministic routing by stochastic forwarding of fragmented key material: each of the *M* fragments is routed by a random walk *variant* from source *s* towards target *t*. Node *t* collects all fragments and derives the final key *block*$\left\{K_{i}\right\}$ of size θ via seeded privacy amplification. We find that the random walk (RW) *variant* plays a significant role in *exposure* $\chi_{s,t}$—the probability of traversing some malicious node, if cartel is positioned optimally for the given (s,t) pair. Building on the QKD transport model from Section 2, we now switch to the algorithmic abstraction. We represent the QKD network as a simple, undirected graph G=(V,E), where *V* denotes the set of trusted nodes and *E* denotes the set of QKD links. The per-link keys $l_{u,v}$ introduced earlier are treated here simply as the hop-by-hop OTP resource available on edge (u,v). To simplify the notation in the analysis, we assume that both the link-key blocks and the derived end-to-end key have lengths of 256 bits. We assume a reactive (rather than a proactive) model. In reactive mode, key *allocation* request triggers a *transmission*, and transmissions are not initiated ahead of time. A transmission (node s→ node *t*) is a key-relaying session by which the final key *block* K of size θ is established. In the base model (without loop erasure), the source *s* emits *M* raw fragments $f_{1},\ldots ,f_{M}$, which eventually reach destination *t* by means of random walks. Later (Section 3.4 and Section 4.2), we adopt τ=98% as the default design threshold (Table 1). At M=1024, the binomial lower bound is $g_{99.99\%}^{★}\left(1024,98\%\right)=6$, which after Theorem 1’s privacy-amplification penalty (with $\varepsilon_{\mathrm{PA}}=2^{-256}$) corresponds to $\theta =g^{★}-2=4$ extractable 256-bit keys per block. Consequently, the ITS-oriented evaluation at τ=98% uses M=1024; smaller fragment counts are only meaningful for this theorem when paired with a lower validated exposure threshold.

### 3.1. Threat Model and Objectives

For information-theoretic security (confidentiality), the adversary controls a cartel C⊆V∖{s,t} of compromised relays. Compromise reveals each relay’s full internal state and the plaintext of every fragment that traverses any relay in *C*. A fragment is treated as compromised once its realized walk intersects *C*. Public-key wrapping of fragments does not satisfy the information-theoretic goal of QKD and is not considered in this paper. We assume honest endpoints (*s* and *t*), authenticated classical channels based on PKI and mutual TLS, and trusted QKD hardware, excluding side-channel attacks [45]. Theorem 1 should therefore be read as a confidentiality theorem under authenticated transport and successful protocol completion, not as an availability guarantee against Byzantine relays. Let κ(s,t) denote the maximum number of internally node-disjoint *s*–*t* paths, equivalently the minimum size of an *s*–*t* vertex cut. The universal ITS target for a pair (s,t) is protection against every relay cartel of size

|C|≤κ(s,t)−1.

This is the strongest possible topology-independent cartel-size target for that pair: a cartel of size κ(s,t) placed on a minimum *s*–*t* cut can intercept every *s*–*t* route. For cartels larger than κ(s,t)−1, we do not claim a universal ITS guarantee. The design parameter for random flow is the exposure threshold τ. For an admissible cartel class $C_{s,t}$ and a realistic topology family, the intended parameter choice must satisfy

$$\max_{C\in C_{s,t}}p_{C}^{s,t}\leq \tau ,$$

where $p_{C}^{s,t}$ is the probability that one random-flow fragment intersects *C*. The value τ is therefore not a universal constant of the protocol. It must be chosen high enough for the evaluated topology and cartel class, and is then used in the binomial lower bound $g_{\alpha }^{★}\left(M,\tau \right)$ and in the privacy-amplification output length. If the measured worst-case cartel exposure on a topology is above 98%, then τ=98% is not a valid ITS design point for that topology and cartel class. In particular, achieving the κ(s,t)−1 target on GÉANT may require experiments at τ>98%. In parallel, we also evaluate a placement-specific non-cut cartel class for |C|≤3. Here, *C* is admissible for a given (s,t) only when *s* and *t* remain connected in G−C. This non-cut condition means that at least one uncompromised *s*–*t* path still exists, even when a particular non-cut placement has size above the universal all-placements target κ(s,t)−1. For these non-cut placements, the ITS claim is again conditional on the measured cartel exposure satisfying $p_{C}^{s,t}\leq \tau$ and on the fragment independence model used by $g_{\alpha }^{★}\left(M,\tau \right)$. Choosing τ below the true exposure invalidates the ITS parameter claim because it overstates the lower bound on full-entropy fragments. It does not automatically imply total key disclosure. If at least one full-entropy fragment avoids the cartel, the construction retains the strictly non-ITS computational fallback described in Section 3.5. This fallback is a residual-confidentiality statement based on a preimage-resistant hash or KDF over the received fragment string, not an information-theoretic guarantee. A compromised relay may also drop, delay, misroute, or inject traffic. We treat such active behavior as an availability and integrity issue, not as part of the ITS confidentiality proof. Drop and delay are availability problems and may cause the transmission to fail to complete. Misrouting is observable through the route metadata reported by the destination and can be flagged at the application layer. Injected or modified traffic is rejected by authenticated classical-channel mechanisms, for example per-hop MACs or AEAD tags derived from link material, together with end-to-end route validation where applicable. The expected outcome of any such active misbehavior, when detected, is rejection or abortion of the transmission rather than silent confidentiality loss. Equivalently, an attacker who can disable QKD links should only be able to reduce availability for a given s→t transmission, not improve its confidentiality advantage beyond the configured exposure model. The design goals are:

1. Information-theoretic security for admissible cartels whose exposure is bounded by τ.
2. Exposure upper bound τ calibrated for realistic medium-scale QKD networks.
3. No per-transmission advantage for attackers who can disable relay links and nodes.
4. Independent stochastic forwarding that utilizes partial disjointness for security.
5. Compatibility with ETSI GS QKD 014 key delivery API.

Forwarding decisions use neighbor information and variant-specific token state; the node-coloring variant introduced below additionally assumes that the source knows the global node-identifier universe. The topology itself may change over time. We prove the ITS statement for the admissible cartel class through the number of realized fragments that avoid the worst admissible cartel. Most realistic mid-term QKD networks are expected to remain sparse because of the high cost of QKD equipment, deployment, and operation. As a result, pairwise connectivity is often low, and the achievable cartel tolerance is inherently limited by min-cut constraints. For biconnected pairs, κ(s,t)≥2, so the universal target always includes at least one compromised relay and grows with the pair’s actual vertex connectivity. Current QKD relay nodes are typically deployed as “trusted nodes” in physically protected facilities, such as telecom points of presence, or secured data centers. Their attack surface is narrower, physical access is controlled, and operational procedures are usually stricter. Consequently, compromise is more likely to arise from a configuration error, insider misuse, supply-chain weakness, or a single negligent administrator. From an operational perspective, multiple simultaneous compromises of relay nodes would represent a high-impact security incident that network operators are strongly motivated to detect and contain quickly. Since QKD networks are expensive, specialized infrastructures with relatively few nodes, they are likely to receive closer monitoring and tighter administrative control than commodity networks. If malicious nodes are not placed optimally on a minimum cut [46] for a given source–target pair, a larger non-cut cartel may still leave enough unseen fragments for ITS under the same τ-bounded exposure condition.

### 3.2. Random Walk Notation

We represent each in-flight (travelling from *s* to *t*) fragment $f_{i}$ by a *token*. Concretely, transmission token *i* is a tuple consisting of id —transmission identifier, source and target pair (s,t), *i*—token index, $f_{i}$—fragment itself, and $\sigma_{i}$—variant-specific token-local state. State $\sigma_{i}$ is the only element that may change over time.

$${\mathrm{token}}_{i}=\left(\mathrm{id},s,t,i,f_{i},\;\sigma_{i}\right),$$

Each token *i* is forwarded by a discrete-time walk. The trajectory of token *i* during the sampled walk is $X_{0},X_{1},\ldots ,X_{h}$, with $X_{0}=s,\;X_{k+1}\in N\left(X_{k}\right)\;,\;X_{h}=t$, where $N\left(X_{k}\right)$ is the neighborhood of $X_{k}$. The whole trajectory is a sampled random sequence ${\left(X_{k}\right)}_{k=0}^{h}$, where *h* is the hop count. The probability distribution from which $X_{k+1}$ is sampled is $P_{\mathrm{variant}}$.

$$X_{k+1}∼P_{\mathrm{variant}}\;\left(\;\;\cdot \;\;|\;X_{k},\sigma_{k}\right),$$

It depends only on the current token *i* and variant-specific state σ. Relays are therefore stateless under this objective and under our interpretation of the random-walk setting. Different fragments use independent forwarding randomness, including independent diversification seeds for HS, so that the exposure events used by the extraction analysis are modeled as independent. The dot in $P_{\mathrm{variant}}\;\left(\;\;\cdot \;\;|\;\ldots \right)$ is a placeholder, and altogether the expression means “distribution over possible next nodes”. The walk terminates when it first hits the target. If the target *t* is an adjacent neighbor of the current relay *v*, i.e., t∈N(v), the relay forwards directly to *t* instead of sampling another stochastic hop. This direct-target rule is the model used in Section 4. For readability, we will adopt the notation $P\;\left(\;v\to u\;|\;★\;\right)$ instead of $P(X_{k+1}\;|\;X_{k},\sigma_{k})$, where $v=X_{k},\;u=X_{k+1}$ and ★ is syntactically substituted for some predicate that can be evaluated from $\sigma_{k}$. It is the probability to go from *v* to *u* given position $X_{k}$ and state $\sigma_{k}$.

### 3.3. Base Random Walk Variants

**Simple random walk (R).** The simple random walk variant R is memoryless: at node *v*, the token chooses the next hop uniformly at random.
  $$P_{R}\left(v\to u\right)=\left\{\begin{array}{cc} 1/|N(v)|, & u\in N(v),\\ 0, & \mathrm{otherwise}. \end{array}\right.$$
  **Non-backtracking random walk (NB).** Non-backtracking NB suppresses immediate return. The token state σ carries a single field prev∈V∪{null} with the previous node (or null at the start). Let $N^{\prime }\left(v\right)=N\left(v\right)\setminus \left\{p\right\}$, where p=prev. Then,
  $$P_{\mathrm{NB}}\left(v\to u\;|\;p\right)=\left\{\begin{array}{cc} 1\;/\;|N^{\prime }\left(v\right)|, & u\in N^{\prime }\left(v\right),\\ 1, & u=p\;\mathrm{and}\;d(v)=1,\\ 0, & \mathrm{otherwise}. \end{array}\right.$$
  After choosing *u*, the token updates prev←v. On regular expander graphs, NB can “mix” provably faster than R [47]. Informally, an *expander* is a sparse graph with strong connectivity.
**Least-recently-visited walk (LRV).** LRV biases the walk away from recently visited vertices. We use an LRV-*vertex* rule: token *i* maintains timestamps last:$V\to N_{0}$, where last[x] is the most recent time at which the token visited *x*. We initialize last[s]=1, and return last[x]=0 by default. At step *k* with $X_{k}=v$,
  $$X_{k+1}\in \arg\min_{u\in N(v)}\mathrm{last}\left[u\right],$$
  breaking ties uniformly. Unvisited neighbors (with value 0) are preferred. Local LRV-type policies are well studied in graph exploration; they can improve practical coverage [48].

### 3.4. Safe Fragment Count Estimation

Because compromised relays may eavesdrop on raw key material, the source emits *M* fragments and estimates how many remain safe under the exposure model below. Let $\chi_{s,t}$ denote the pair-specific single-relay *exposure*: the max probability that the adversary, placed on the worst admissible intermediate relay, observes a fragment travelling s→t. Because nodes do not know the topology in advance, they fix a design threshold τ and choose *M* from $g_{\alpha }^{★}\left(M,\tau \right)$, requiring $\chi_{s,t}\leq \tau$ for every pair (s,t) in the relevant admissible cartel class. The admissible value of τ depends greatly on the random walk variant. For a single relay *v* or an unordered cartel of *m* intermediate relays C⊆V∖{s,t} with |C|=m, define the hit probability

$$p_{C}^{s,t}\;:=\;\mathrm{Pr}\left[X\cap C\neq \emptyset \right].$$

Let [A] denote the indicator of a condition *A*, equal to 1 when *A* is true and 0 otherwise. Empirically, for *W* sampled walks $X^{\left(1\right)},\ldots ,X^{\left(W\right)}$, the cartel-hit probability is estimated as follows:

$${\hat{p}}_{C}^{s,t}=\frac{1}{W}\sum_{i=1}^{W}\left[\;X^{\left(i\right)}\cap C\neq \emptyset \;\right].$$

Equivalently, ${\hat{p}}_{C}^{s,t}$ is the fraction of walks on which at least one relay in *C* is visited. For computation speed-up, the same union-hit count can be computed from marginal and intersection counts by inclusion–exclusion. For relays u,v∈C let $N_{u}$ be the number of walks visiting *u* and $N_{u,v}$ the number visiting both *u* and *v*; for |C|=2,

$$\left|\{i:X^{\left(i\right)}\cap C\neq \emptyset \}\right|=N_{u}+N_{v}-N_{u,v},$$

and for |C|=3 the same idea extends to $N_{a}+N_{b}+N_{c}-N_{a,b}-N_{a,c}-N_{b,c}+N_{a,b,c}.$ We denote the corresponding worst-case *m*-relay exposure over non-cut placements by

$$\begin{array}{c} \chi_{s,t}^{\left(m\right)}\;:=\;\max_{C\in D_{s,t}^{\left(m\right)}}p_{C}^{s,t},\;\chi_{s,t}\;:=\;\chi_{s,t}^{\left(1\right)}.\\ D_{s,t}^{\left(m\right)}:=\left\{C\subseteq V\setminus \{s,t\}:|C|=m,\;s\;\mathrm{and}\;t\;\mathrm{remain}\;\mathrm{connected}\;\mathrm{in}\;G-C\right\} \end{array}$$

**Figure 3:**
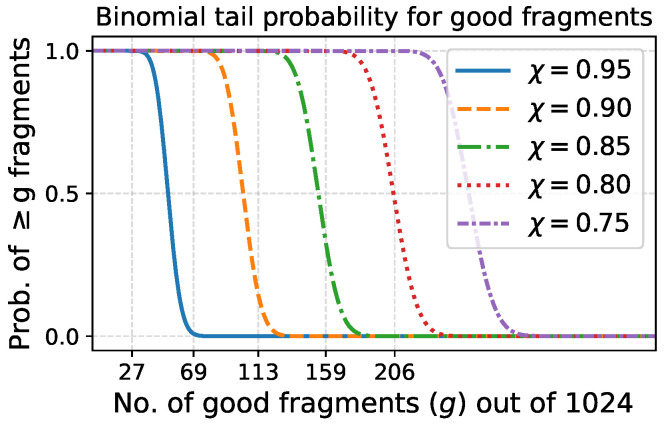
Probability of receiving at least *g* safe fragments.

For |C|=1, a relay *v* lies in $D_{s,t}^{\left(1\right)}$ if removing *v* does not disconnect *s* from *t*. Assume we transmit *M* fragments and let *G* denote the number of *safe* fragments, i.e., fragments not observed by the compromised relay or relay cartel. Then, Pr[G≥g] follows a cumulative binomial distribution (Figure 3) and can be calculated as follows:

Pr[G≥g]=∑k=gMMk1−χkχM−k

Let us define $g_{\alpha }^{★}\left(M,\chi \right)$ as the largest *g* such that Pr[G≥g] is at least, e.g., α=99.99%. It is a statistical lower bound on the number of fragments that retain full entropy in transmission s→t, with failure probability at most 1−α under the independent-fragment exposure model and the chosen value of $\chi_{s,t}$.

$$g_{\alpha }^{★}\left(M,\chi \right)\;=\;\max\left\{\;g\in \{0,\ldots ,M\}:\mathrm{Pr}[G\geq g]\geq \alpha \;\right\}$$

**Figure 4:**
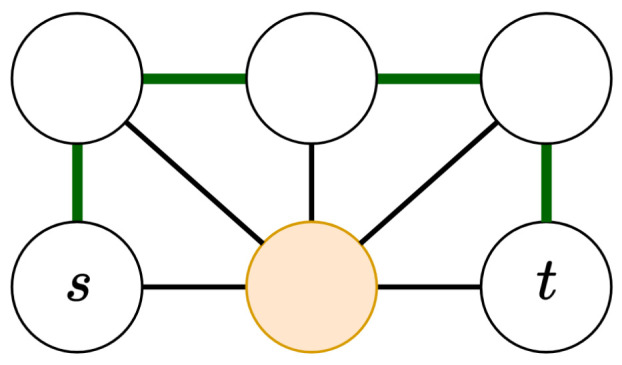
High χ construction example. Assume the yellow node at the bottom, between *s* and *t*, is compromised. The green line indicates the remaining safe path from *s* to *t*. Along this path, at each *v*, the correct neighbor must be chosen.

See Table 1 for $g_{\alpha }^{★}$ values corresponding to different assumed χ values. For M=1024 and χ=98%, the table gives $g_{99.99\%}^{★}\left(1024,98\%\right)=6$. We take τ=98% as the default design threshold. On the synthetic 99-node graph, the worst-case HS single-relay exposure is 93.3% (Figure 4), so the recommended τ exceeds the observed single-relay exposure there; multi-node cartel coverage at this threshold is evaluated in Section 4.7. On the 43-node GÉANT topology, the same threshold also upper-bounds the worst-case HS exposure (91.3%). For a fully conservative calculation on another topology, one should use that topology’s observed or assumed HS design exposure instead. The $g_{\alpha }^{★}$ calculation is deliberately conservative because it sets the fragment-count parameter *M* used in the information-theoretic extraction claim of Section 3.7.

### 3.5. Computational Fallback

The exposure threshold τ determines whether the chosen output length θ has the information-theoretic extraction guarantee of Theorem 1. It does not by itself determine whether the transmission is completely disclosed. If the realized number of unseen full-entropy fragments is positive but below the lower bound needed for the configured θ, the leftover-hash guarantee for that θ no longer applies, but the adversary still has not learned the whole received fragment string. This motivates a weaker, strictly non-ITS fallback. In addition to seeded privacy amplification, an implementation can also pass the full received fragment string through a preimage-resistant 256-bit hash or KDF, such as SHA3-256 or HKDF. An adversary who has observed all but at least one full-entropy 256-bit fragment then faces a preimage/search problem over the unseen fragment bits; in the idealized model, Grover search leaves roughly $2^{128}$ quantum work for a 256-bit unknown [49]. This is not an information-theoretic claim, and the evaluation tables do not count it as ITS output. It only states residual computational confidentiality when at least one fragment escapes the compromised relay or relay cartel. Under the independent-fragment exposure model, the probability that no fragment escapes is $\chi^{M}$. Equivalently, over *N* independent transmissions, the expected number of transmissions with complete fragment disclosure is $N\chi^{M}$. For the default M=1024, exposure χ=98% is therefore a useful reference point: $10^{9}\;\cdot \;0.98^{1024}\approx 1.04$. Thus, at roughly one billion transmissions, an exposure of 98% corresponds to about one expected complete-disclosure event, while lower exposure gives a rapidly smaller rate. This is the main reason for keeping *M* large even when the ITS-oriented extracted block θ is small: a high fragment count sharply suppresses the probability that a high-exposure relay placement observes every fragment. There is a separate defense-in-depth fallback at the application layer. As discussed in Section 2, the communicating applications can run their own post-quantum or hybrid key exchange and combine it with QKD-derived material. That endpoint mechanism does not rely on trusting the QKD relay network, whereas the fallback in this subsection is the residual confidentiality left by random flow itself when at least one fragment remains hidden.

### 3.6. Realized-Path Cartel Accounting

The binomial value $g_{\alpha }^{★}$ is a conservative ex-ante lower bound on the number of safe fragments. If verified realized paths are available, the destination can instead compute the safe-fragment count directly from those paths. How to make the destination cryptographically confident in the reported paths is outside this paper’s scope and left as future work. We only record the accounting that such a mechanism would enable. For every possible compromised relay, count how many received fragments did *not* pass through that relay. The adversary is assumed to choose the relay that gives the smallest such count, so the exact worst-case single-relay count is

$${\hat{g}}_{s,t}^{\left(1\right)}=\min_{v\in D_{s,t}^{\left(1\right)}}\left|\{i:v\notin X^{\left(i\right)}\}\right|.$$

For an *m*-relay cartel, we perform the same calculation over candidate relay sets: for each cartel $C\in D_{s,t}^{\left(m\right)}$, count the fragments whose paths avoid all relays in *C*, then take the smallest count. The corresponding value is

$${\hat{g}}_{s,t}^{\left(m\right)}=\min_{C\in D_{s,t}^{\left(m\right)}}\left|\{i:X^{\left(i\right)}\cap C=\emptyset \}\right|.$$

The minimum is necessary because the adversary is placed in the worst candidate relay or cartel, matching the definition of exposure as a maximum hit probability. Define the realized-path safe-fragment yield as $\rho_{s,t}^{\left(m\right)}:={\hat{g}}_{s,t}^{\left(m\right)}/M$. Under the independent-fragment exposure model, for the universal target m=κ(s,t)−1,

$$\rho_{s,t}^{\left(m\right)}\underset{M\to \infty }{\to }1-\chi_{s,t}^{\left(m\right)}.$$

After the two-fragment privacy-amplification penalty below, the extracted-key yield $\rho_{s,t}^{\mathrm{ext}}:=\theta_{s,t}/M$ has the same asymptotic limit.

### 3.7. Privacy Amplification

Let $\theta_{s,t}$ denote the number of final 256-bit keys extracted after privacy amplification for transmission s→t. Then, $g_{\alpha }^{★}$ should be understood as a lower bound on the number of safe fragments, not directly on $\theta_{s,t}$. Since verified realized paths are left to future work, the default privacy-amplification parameter is derived from the configured design threshold τ:

$$g_{\tau }:=g_{\alpha }^{★}\left(M,\tau \right),\;\theta_{\tau }:=\max\left(0,g_{\tau }-2\right).$$

Here, $g_{\tau }$ is a high-confidence lower bound on the number of safe fragments under the independent-fragment exposure model and the condition that every admissible cartel has per-fragment exposure at most τ. If verified realized paths become available, the exact count $\hat{g}$ from Section 3.6 can replace $g_{\tau }$. At the destination, the recovered fragment material is concatenated into *Z* and processed by a seeded universal-2 extractor, $K={\mathrm{Ext}}_{S}\left(Z\right),$ where the seed *S* is public but authenticated end to end. For an ITS claim, this step should use a privacy-amplification construction covered by the Leftover Hash Lemma, for example a universal-hash implementation such as Toeplitz hashing [50]. It should not be replaced by an ordinary cryptographic hash function when the claim being made is information-theoretic security. Cryptographic hashes or KDFs only support the computational fallback discussed in Section 3.5. Given $g_{\tau }$, we can state the following theorem.

**Theorem** **1.** *Assume that, for transmission s→t, each admissible relay cartel observes any one fragment with probability at most τ, and fragment exposure events are independent across fragments. Let $g_{\tau }=g_{\alpha }^{★}\left(M,\tau \right)$. Then seeded universal-2 privacy amplification with $\varepsilon_{\mathrm{PA}}=2^{-256}$ extracts $\theta_{\tau }=\max\left(0,g_{\tau }-2\right)$ full 256-bit keys that are $\varepsilon_{\mathrm{PA}}$-close to uniform except with probability at most 1−α under the stated exposure model. No extraction occurs when $g_{\tau }\leq 2$.*

**Proof.**  Fix the public transcript (extractor seed and any public protocol messages) and an admissible adversary A. By the definition of $g_{\tau }=g_{\alpha }^{★}\left(M,\tau \right)$, the event that A misses at least $g_{\tau }$ of the *M* fragments has probability at least α under the independent-fragment exposure model. Condition on this event. Each fragment $f_{i}\in {\left\{0,1\right\}}^{256}$ is sampled at the source uniformly and independently of all other fragments and of the public transcript. Therefore, conditioned on the public transcript and A’s view of the at most $M-g_{\tau }$ fragments it observes on this event, the remaining $g_{\tau }$ unseen fragments are jointly uniform on ${\left\{0,1\right\}}^{256g_{\tau }}$. The conditional min-entropy of the *M*-fragment string given A’s view is therefore at least $256\;g_{\tau }$ bits. By the Leftover Hash Lemma with a universal-2 hash family and an independent public seed [51,52,53], privacy amplification extracts at most

$$l\leq 256\;g_{\tau }-2\log_{2}\left(1/\varepsilon_{\mathrm{PA}}\right)$$

bits whose distribution is $\varepsilon_{\mathrm{PA}}$-close to uniform conditioned on A’s view. Flooring to whole 256-bit keys gives

$$\theta_{\tau }=\max\left(0,\left\lfloor \frac{256\;g_{\tau }-2\log_{2}\left(1/\varepsilon_{\mathrm{PA}}\right)}{256}\right\rfloor \right).$$

For $\varepsilon_{\mathrm{PA}}=2^{-256}$, this reduces to $\theta_{\tau }=\max\left(0,g_{\tau }-2\right)$. The seed can be public because LHL is a strong extractor; in our setting, it is transmitted over the authenticated classical channel so that source and destination derive the same keys. The event $G\geq g_{\tau }$ fails with probability at most 1−α, and any residual error in the assumed exposure bound τ contributes an additional term $\varepsilon_{\mathrm{est}}$. By a union bound, the total distinguishing advantage between the extracted key and an ideal uniform key, conditioned on the public transcript and adversarial view, satisfies

$$\varepsilon_{\sec}\leq \left(1-\alpha \right)+\varepsilon_{\mathrm{PA}}+\varepsilon_{\mathrm{est}}.$$

   □

### 3.8. Exposure Reduced RW Variants

The simulation results in Section 4.2 show that the fully local variants R, NB, and LRV have high $\max_{s,t}\chi_{s,t}$ values. On each evaluated graph, there exists a pair s,t such that $\chi_{s,t}\geq 96.4\%$ for each of these walk variants. See Figure 4 for intuition about why high χ can arise. There is often a longer bypass path connecting *s* and *t*, but the walk is likely to be diverted back into the more “congested” direct connection. To address this, we can temporarily color nodes one by one, or otherwise assign them lower priority throughout the walk. Different walks within the same transmission should penalize different nodes. The following constructions reduce maxχ from 95.3% (LRV) to 91.3% (HS). The expected number of fragments not observed by single-relay cartel is proportional to 1−χ. It therefore increases by 85.1% because 1−95.3=4.7%, and 1−91.3=8.7%.

**One-by-one node-coloring (NC).** If *s* and *t* are biconnected, it is possible to exclude nodes one-by-one from the graph, by coloring (marking) them. That still keeps connectivity between *s* and *t* but forces the random walk to avoid the excluded vertex, thus guaranteeing that it will not know at least one fragment. This approach, however, requires the node-identifier universe *W* from which excluded relays are selected. Thus, NC should be described as *partially topology-oblivious*. Nevertheless, the NC variant does not require adjacency knowledge, or path computation. If M>|W|, we can color vertices in circular order (thus, some nodes will miss several fragments). Otherwise, when choosing the next hop, we apply the LRV walk strategy, taking into a consideration the colored (excluded) node.

**Theorem** **2.** *In the presence of a single malicious node, the NC random walk variant guarantees at least one key that is uniform under this idealized secret-sharing model.*

**Proof.**  This is the standard additive (XOR) secret-sharing argument [54]. For $K\in {\left\{0,1\right\}}^{256}$, sample $f_{1},\ldots ,f_{M-1}$ uniformly and set $f_{M}=K⊕{⨁}_{i=1}^{M-1}f_{i}$, so $K={⨁}_{i=1}^{M}f_{i}$. If the compromised relay misses at least one fragment, then the XOR of the fragments it misses is still uniform over ${\left\{0,1\right\}}^{256}$ and independent of the fragments it sees. XORing this unknown uniform value with the relay’s fixed view leaves *K* uniform from that relay’s perspective.    □

Although additive (XOR) secret sharing is easy to implement and reason about, we instead quantify χ and apply entropy extraction (as in Theorem 1) for higher yield ρ.

**Figure 5:**
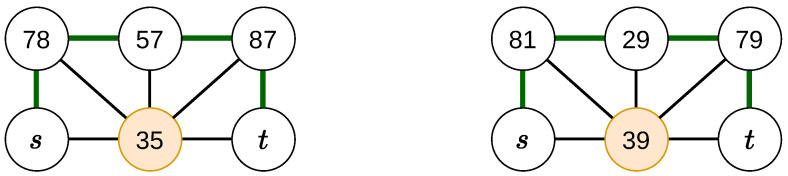
Two HS score assignments on the same topology. Node colors indicate the per-seed score ordering, and the highlighted lines indicate the selected next-hop choices. In the first run, the central relay has the lowest score and is avoided; in the second, it has a higher score than one of the nodes and is selected.

**Highest-score neighbor (HS).** HS is a seed-based local diversification heuristic. For each fragment token *i*, the source samples a fresh seed $\zeta_{i}\overset{\$}{\leftarrow }{\left\{0,1\right\}}^{\lambda }$. The seed gives each vertex *u* a deterministic per-token score
  $${\mathrm{score}}_{i}\left(u\right)=h\left(u,\zeta_{i}\right),$$
  where *h* is a deterministic mixing function of the vertex identifier *u* and seed $\zeta_{i}$. Different seeds induce different local rankings without requiring topology knowledge. Thus, a high-exposure relay can receive a low score for some fragments. Let $U_{i}\left(v\right)\subseteq N\left(v\right)$ be the neighbors not yet visited by token *i*. If $U_{i}\left(v\right)$ is nonempty, HS chooses a highest-scoring unvisited neighbor, breaking ties uniformly:
  $$X_{k+1}^{\left(i\right)}\in \arg\max_{u\in U_{i}\left(v\right)}{\mathrm{score}}_{i}\left(u\right)$$
  If all neighbors have already been visited, HS falls back to the NB rule. Operationally, a relay computes these scores only for its current neighbors and for the current token seed $\zeta_{i}$. No relay stores a persistent score table; the token carries only its seed, previous hop, and visited-node set. Figure 5 illustrates how different per-fragment seeds can rank the same relay differently and thereby diversify exposure.

### 3.9. Efficiency and Throughput

For a fixed source–destination pair (s,t), let $H_{s,t}$ denote the random hop count of one fragment walk, and let $h_{s,t}\;:=\;E\left[H_{s,t}\right]$ be the expected hop count. As previously assumed, the fragment size is 256 bits and matches link key size. One delivered fragment therefore consumes, in expectation, $h_{s,t}$ link keys. A transmission consumes $M\;\cdot \;h_{s,t}$ link keys. Suppose a transmission uses *M* fragments. Under the independent-fragment exposure model, the expected fraction of fragments that avoid a worst-case admissible cartel is the complement of the corresponding cartel exposure. Let m:=κ(s,t)−1. For a fixed design threshold τ on admissible cartel exposure, define the pair-specific expected yield and the conservative design yield side by side:

$$\rho_{s,t}\;:=\;1-\chi_{s,t}^{\left(m\right)},\;\rho^{\tau }\;:=\;\frac{g_{\alpha }^{★}\left(M,\tau \right)}{M}.$$

Section 3.6 motivates this choice: ${\hat{g}}_{s,t}^{\left(m\right)}/M$ converges to $1-\chi_{s,t}^{\left(m\right)}$, so $\rho_{s,t}$ is the expected safe-fragment count per emitted fragment, not the conservative binomial lower bound $g_{\alpha }^{★}\left(M,\chi \right)/M$ used for ITS parameter selection. Thus, $\rho^{\tau }$ is a high-confidence lower bound on the safe-fragment yield under the design threshold, while $\rho_{s,t}$ is the pair-specific expected yield. We use ρ throughout for safe-fragment yield and reserve θ for the extracted-key count, whose default value is $\theta_{\tau }=\max\left(0,g_{\tau }-2\right)$ from Theorem 1. Because QKD-derived link keys are scarce, we define the *model-based extracted efficiency* as a heuristic proxy for extracted output bits per consumed QKD-derived link-key bit:

$$\eta_{s,t}\;:=\;\rho_{s,t}\;h_{s,t}^{-1},\;\eta_{s,t}^{\tau }\;:=\;\rho^{\tau }\;h_{s,t}^{-1}.$$

This is not an exact extracted-key count: ρ tracks safe-fragment yield before the additive LHL penalty, while exact default counts use $\theta_{\tau }=\max\left(0,g_{\tau }-2\right)$. For the reported M∈{256,1024}, this penalty is at most 0.8% of *M* and is included only in quantitative claims about θ. When reporting a topology-wide metric over a set P of ordered (s,t) pairs, we use   

$$\bar{\eta }\;:=\;\frac{1}{\left|P\right|}\sum_{(s,t)\in P}\eta_{s,t},\;{\bar{\eta }}^{\tau }\;:=\;\frac{1}{\left|P\right|}\sum_{(s,t)\in P}\eta_{s,t}^{\tau }.$$

Next, define the raw fragment throughput $T_{s,t}$ as the long-run average rate, in bits/s, at which fragment bits arrive at the destination *t*. We assume that only one ordered pair (s,t) is active at a time, that QKD links continuously generate and buffer keys (starting with empty buffers). The corresponding model-based extracted throughput is given by

$$R_{s,t}\;:=\;T_{s,t}\;\rho_{s,t},\;\mathrm{equivalently}\;R_{s,t}=T_{s,t}\;\eta_{s,t}\;h_{s,t}.$$

Thus, $\eta_{s,t}$ captures model-based extracted efficiency, while $R_{s,t}$ captures the corresponding extracted-throughput proxy under the simplified entropy model.

### 3.10. Loop Erasure

We separate route discovery from payload transmission. In the first phase, the source emits a lightweight *scouting token* over the authenticated classical channel only. The scouting token carries no secret fragment material and consumes no QKD-derived link-key bits. It is forwarded according to the same stochastic forwarding rule as the corresponding payload walk and records the visited history

$$W=(v_{0}=s,v_{1},\ldots ,v_{\tau }=t).$$

When we later report exposure for loop-erased variants, we still measure it on this original scouting walk as a conservative upper bound on literal payload exposure. During the walk a time-bounded link key reservation is applied to traversed links. Ultimately, if some relay along this sampled walk cannot admit the request, the session may be rejected before any key material is transmitted; in a REST realization, this can be surfaced as an implementation-level failure such as HTTP 503. After the scouting token reaches *t*, the recorded walk *W* is converted into a realized payload route by *chronological loop erasure* [55,56]. Concretely, one scans *W* from left to right and, whenever a vertex reappears, deletes the closed subwalk between the earlier occurrence and the repeat, while retaining a single copy of the repeated vertex. Let LE(W) denote the resulting simple *s*–*t* path. In the second phase, the actual fragment material is forwarded hop by hop along LE(W), with each hop protected by fresh QKD-derived link-key bits used as a one-time pad. Strictly speaking, for the NB, LRV, NC, and HS variants, LE(W) is not the *classical* loop-erased random walk distribution from probability theory; here loop erasure is used only as a path-simplification operator applied to the sampled scouting history. This two-phase construction provides two operational benefits. First, removing loops shortens the realized payload route, thereby reducing expected hop count, QKD key consumption, and queueing pressure. Second, because the realized payload route is simple, self-induced cyclic waiting caused by one fragment revisiting the same relays is eliminated. Rejection, if necessary, occurs before secret material enters the relay path. The interpretation of exposure under loop erasure requires care. A compromised relay that appears only on an erased segment of the scouting walk does not directly observe fragment plaintext, because the scouting phase is classical only. However, such a relay may still influence the realized route by biasing the scouting trajectory or by affecting admission decisions. Therefore, when reporting $\chi_{s,t}$ for loop-erased variants, we count scouting hits as a *conservative upper bound* on literal payload exposure. This preserves comparability with the no-loop-erasure case while avoiding an optimistic estimate of adversarial influence.

### 3.11. Network Dynamism

The topology-oblivious forwarding model does not remove the need for controlled topology changes. Relevant changes include adding a relay or QKD link, removing a relay and its incident links, and removing a QKD link. We assume supervised changes: the administrator of a relay manually registers or unregisters its incident QKD links and neighboring relays. Because the operation is local, the administrator does not need a full network view. Adding QKD links cannot decrease the minimum vertex cut between any fixed source–target pair [46]. It can nevertheless change exposure by making some relays more likely to appear on sampled walks, so exposure should be re-evaluated after topology expansion. Adding a relay can also reduce throughput until the new incident QKD links have enough key material, because pairwise link keys must be established with that relay. If the new relay is later compromised, fragments traversing it no longer contribute full entropy, and the privacy-amplification output length must be reduced according to the updated exposure model. We argue against automatic removal of QKD nodes or links from the forwarding graph. Silent removal can change the security model seen by forwarding relays without updating the configured exposure threshold. For brittle QKD-link failures or exhausted link-key buffers, the safer behavior is fail-stop rejection. If a relay cannot forward using an available QKD link while satisfying the configured threat model and key-availability assumptions, the transmission should fail and the operator should be notified. This reduces availability, but it prevents the implementation from silently routing under an exposure model that no longer matches the configured parameters. Classical-link dynamism is treated separately. The classical transport stack retransmits lost packets and eventually fails after a timeout. Such failures are reported to the operator, who can then reconfigure the QKD-network view if needed.

## 4. Simulation Study

We focus on three questions: (i) *exposure*—how large the worst-case relay hit probability $\chi_{s,t}$ can be for some source–target pair, and what variant-specific worst-case or illustrative design exposure this suggests when choosing the target extracted block size θ and, where applicable, the fragment count *M*; (ii) *efficiency*—what extracted-yield estimators and model-based output per consumed QKD-derived link-key bit the evaluated variants achieve under the paper’s simplified entropy model; and (iii) *scalability*—how these quantities and the routing cost evolve with network size. We address these questions on topologies resembling existing quantum network deployments.

### 4.1. Simulated Topologies

**Figure 6:**
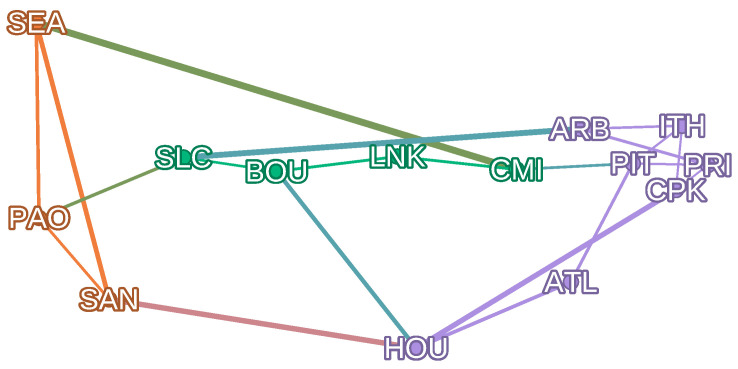
Reconstructed NSFNET T1 topology. Node colors denote Gephi modularity communities and have no physical or administrative meaning.

Trusted-node QKD deployments are currently small (typically tens of nodes) due to the cost of dark fiber and QKD equipment. For context, the largest openly reported QKD deployment is the China Quantum Communication Network (CN-QCN), spanning 145 nodes [57]; however, CN-QCN uses centralized network management and exhibits a hierarchical topology with many articulation points. For our simulation study, we selected two deployed-analog topologies with 14 and 43 nodes (NSFNET and GÉANT). To study scaling trends, we additionally use a synthetic 99-node graph described in Section 4.8. Both topologies are visualized in Gephi 0.10 using a geographic layout based on approximate site latitude/longitude, and node colors denote Gephi modularity communities with no physical or administrative meaning. We deliberately focus on graphs with a nontrivial biconnected structure, and, whenever we report ITS-oriented exposure, yield, efficiency, or throughput quantities, we restrict attention to biconnected ordered pairs (s,t). The NSFNET T1 backbone from 1991 with 14 nodes [58]. Although NSFNET is a classical network, it serves as a medium-sized, historically relevant analog: in early wide-area optical transport, capacity was scarce and expensive, motivating sparse topologies and careful end-to-end provisioning. Similar constraints reappear in QKD networks. Figure 6 shows the reconstructed graph used in the simulations.

**Figure 7:**
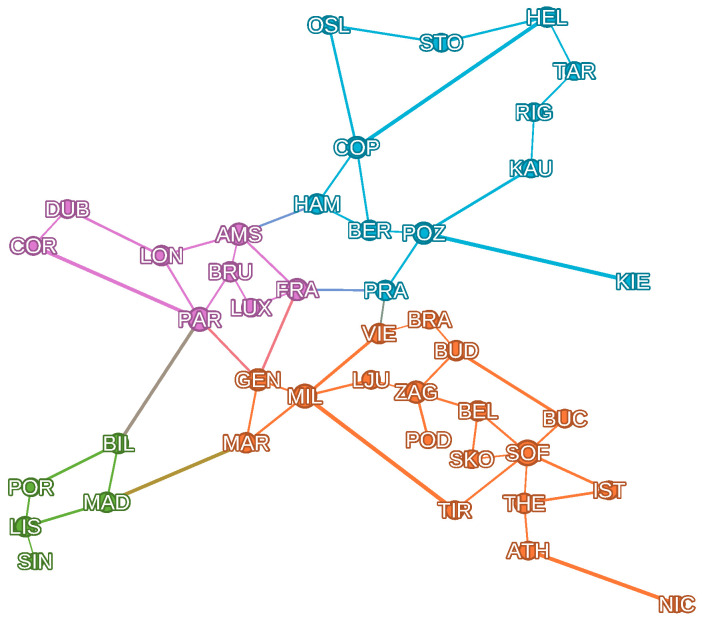
Adapted version of GÉANT GN4-3N. Node colors denote Gephi modularity communities and have no physical or administrative meaning.

The GÉANT GN4 Phase 3 backbone (GN4-3N) is a large, well-connected topology. Our variant, after pruning links longer than 1000 km to improve topology perceptibility in the graph diagram, has 43 nodes and 59 edges. GÉANT is also engaged in Europe’s “ultra-secure” communications direction (including EuroQCI-oriented efforts that consider QKD overlays), making this backbone a plausible substrate for a future QKD overlay [59,60]. Figure 7 shows the adapted GÉANT graph.

Table 2 summarizes basic structural metrics of the two deployed-analog topologies.

In practice, the GÉANT and NSFNET topologies would require many additional relay sites because their longest links exceed the ≈150–300 km range typical of current commercial devices. We do not introduce such relays here; these two graphs are treated as structural proxies. Notice also that, as pointed out in the introduction, dense QKD networks are not expected in the near future.

### 4.2. Single-Node Exposure

To evaluate the security-relevant exposure, we estimate the exposures defined in Section 3.4 by Monte Carlo sampling. Throughout this section, hat notation marks empirical quantities computed from sampled walks; for example, ${\hat{p}}_{v}^{s,t}$ and ${\hat{\chi }}_{s,t}$ are the sampled counterparts of $p_{v}^{s,t}$ and $\chi_{s,t}$. For single-relay exposure, we apply the cartel-hit estimator there with |C|=1, i.e., ${\hat{p}}_{v}^{s,t}:={\hat{p}}_{\left\{v\right\}}^{s,t}$ and ${\hat{\chi }}_{s,t}=\max_{v\in D_{s,t}^{\left(1\right)}}{\hat{p}}_{v}^{s,t}$. We sample W=10,000 loop-erased walks per ordered source–target pair and variant. Each walk uses the run index r∈{0,…,W−1} as its deterministic PRNG seed in the C++ exposure simulator, compiled with g++ 16.1.1 using default compile settings; hop-count experiments use the same convention with W=1000. The synthetic scaling graph is generated with Python 3.12 seed 2026. We report point estimates only: at W=10,000 the Monte Carlo standard error on a 90% hit rate is about 0.3 percentage points, so small differences between close variants should not be overinterpreted. Graph files, reconstruction scripts, and analysis code are available in the accompanying repository. In each graph, we then identify $\max_{s,t}\hat{\chi }$ for each RW variant. For the single-relay admissible class, this worst-case value is the relevant exposure to compare with the design threshold τ when setting *M* and the extracted block size $\theta_{\tau }$ through $g_{\alpha }^{★}\left(M,\tau \right)$. For loop-erased variants, $X^{\left(i\right)}$ still denotes the original sampled/scouting walk rather than only the realized payload route, so the reported $\hat{\chi }$ remains a conservative upper bound on literal payload exposure. Table 3 reports the detailed GÉANT breakdown, including the maximizing (s,t,v) triple and the average $\hat{\chi }$ over all ordered pairs, while Table 4 summarizes per-graph max and median $\hat{\chi }$ across variants. On GÉANT, NC exceeded the walk step limit on 530 ordered pairs; those pairs are omitted from the NC aggregates in Table 3 and Table 4, which is one reason NC is treated as a diagnostic variant rather than an implementer-facing recommendation.

As reported in Table 3 and Table 4, HS attains the lowest worst-case exposure on NSFNET, and also the lowest worst-case exposure on GÉANT under the non-cut single-relay metric. The reported maximum is a max-of-max quantity: for each ordered source–target pair, the relay position is chosen adversarially within $D_{s,t}^{\left(1\right)}$, and the largest such value is then reported over all pairs. On GÉANT, the worst eligible single relay for HS is PAR on the MAD–COR pair, giving $\hat{\chi }=91.3\%$. The median exposure is lower (71.7% for HS), meaning that for a typical ordered pair, even the best-positioned non-cut compromised relay misses roughly a quarter of the fragments. Among the evaluated variants, HS gives the best worst-case exposure. NC remains close on GÉANT, but it assumes the globally known node set, whereas HS preserves the stronger locality of using only neighbor information plus token state.

### 4.3. Multi-Node Exposure

We also use the same empirical exposure calculation to evaluate two- and three-node compromised relay cartels *C*. Call an ordered pair (s,t) *eligible* for cartel *C* if s,t∉C and *s* and *t* remain connected in the subgraph G[V∖C] obtained by removing all cartel nodes—equivalently, *C* is not an *s*–*t* vertex cut, so at least one *C*-disjoint *s*–*t* path exists in G[V∖C] and a topology-aware multipath baseline (Section 4.7) could in principle route safely between them. Formally,

$$E\left(C\right)\;=\;\left\{(s,t):s,t\notin C,\;s\;\mathrm{and}\;t\;\mathrm{are}\;\mathrm{connected}\;\mathrm{in}\;G[V\setminus C]\right\}.$$

A cartel is itself called *eligible* if |E(C)|>0, i.e., it does not disconnect every source–target pair. Call an eligible pair (s,t) *affected* at design threshold τ if its empirical cartel exposure exceeds that threshold:

$$A\left(C,\tau \right)\;=\;\left\{\left(s,t\right)\in E\left(C\right):p_{C}^{s,t}>\tau \right\}.$$

An affected pair is one for which the chosen threshold τ would overestimate the number of full-entropy output keys for that cartel. This does not necessarily mean that every fragment is exposed: as discussed in Section 3.5, if a cartel’s per-fragment exposure is $\chi_{C}<1$, then the independent-fragment model gives probability $\chi_{C}^{M}$ that the cartel observes all fragments. For example, $\chi_{C}=99\%$ and M=1024 gives $0.99^{1024}\approx 0.0034\%$. Hence, even when a cartel invalidates the configured ITS parameter choice, the strictly non-ITS computational fallback discussed in Section 3.5 (preimage-resistant 256-bit hash or KDF over the received fragment string) still applies whenever at least one full-entropy fragment is not observed. This is not the information-theoretic claim; it is only a computational fallback when at least one full-entropy fragment remains hidden. In the idealized model, Grover search leaves roughly $2^{128}$ quantum work for a 256-bit unknown [49]. Table 5 reports this GÉANT HS analysis for two- and three-node cartels. For each candidate design threshold τ and cartel size, it gives the median, average, and maximum of |A(C,τ)|/|E(C)| over all eligible unordered cartels *C*.

These summary statistics should be interpreted descriptively: most two- or three-node cartels are not chosen to target a specific source–target pair and are therefore placed rather arbitrarily with respect to any given (s,t). At the default τ=98%, the average affected-pair fraction is 0.00% for two-node cartels and 0.02% for three-node cartels. The maximum column is nevertheless useful as a warning about favorable placements for a relay cartel: the worst eligible two-relay cartel affects no eligible ordered pairs at τ=98%, while the worst eligible three-relay cartel affects 19.49%. Thus, the single-relay exposure results should not be read as a multi-relay guarantee, even though a computational fallback remains when at least one fragment escapes the cartel.

### 4.4. Expected Hop Count

To put these numbers into context, they remain well above the corresponding average shortest-path lengths on NSFNET and GÉANT. The longest mean path after loop-erasure is attained by HS. This aligns with its original path-diversification motivation.

### 4.5. Efficiency

From Section 3.9, pairwise efficiency factors as $\eta_{s,t}=\rho_{s,t}\;h_{s,t}^{-1}$ with $\rho_{s,t}=1-\chi_{s,t}^{\left(\kappa (s,t)-1\right)}$. We further distinguish *pair-specific* efficiency from *design-threshold* efficiency $\eta_{s,t}^{\tau }=\rho^{\tau }h_{s,t}^{-1}$ with $\rho^{\tau }=g_{\alpha }^{★}\left(M,\tau \right)/M$. For the pair-specific case, we write

$$\eta_{s,t}^{\hat{\chi }}:=\left(1-{\hat{\chi }}_{s,t}^{\left(m\right)}\right)\;h_{s,t}^{-1},\;m:=\kappa \left(s,t\right)-1,$$

where ${\hat{\chi }}_{s,t}^{\left(m\right)}$ is the measured (κ(s,t)−1)-relay cartel exposure for (s,t). For ${\bar{\eta }}^{\tau }$, we set τ to the graph- and variant-specific worst-case single-relay exposure $\max_{s,t}\hat{\chi }$ from Section 4.2 (Table 4). On NSFNET, these thresholds range from 71.0% (HS) to 78.1% (NB); on GÉANT, from 91.3% (HS) to 95.7% (NB). In the simulation results below, we report topology-wide averages ${\bar{\eta }}^{\tau }$ and ${\bar{\eta }}^{\hat{\chi }}$ over all biconnected ordered pairs. Since exposure is still computed from the original walk, loop-erasure affects only the denominator through the expected hop count. In Table 6 and Table 7, we omit the simple random walk R and focus on NB, LRV, NC, and HS, since R is already dominated on the evaluated graphs in both exposure and expected hop count. Table 6 shows that the design-threshold efficiency substantially understates the pair-specific efficiency on all evaluated topologies: ${\bar{\eta }}^{\hat{\chi }}$ is consistently far above ${\bar{\eta }}^{\tau }$. The reason is that ${\bar{\eta }}^{\tau }$ fixes one worst-case exposure per graph and variant, whereas ${\bar{\eta }}^{\hat{\chi }}$ uses the exposure measured for each source–target pair. Within each metric row, Table 6 also compares original walks with loop-erased routes. The right block uses the loop-erased routes, and the left block uses the original sampled walks. The resulting increase in efficiency is noticeably smaller than the corresponding reduction in mean hop count from Table 8. This is not a contradiction. Although loop-erasure reduces average hop count dramatically, efficiency scales with the reciprocal hop count $h^{-1}$, not with *h* itself. Thus loop-erasure primarily removes a heavy tail of excessively long walks, rather than uniformly increasing $h^{-1}$ for all source–target pairs.

The distribution of pairwise efficiencies is strongly right-skewed, especially on GÉANT. For instance, for NB without loop-erasure on GÉANT, the mean ${\bar{\eta }}^{\hat{\chi }}$ is 9.7% whereas the median is only 0.72%; with loop-erasure, the corresponding values are 12.1% and 3.1%. Thus, the mean efficiency should be read as a topology-wide average, not as a typical per-pair value.

### 4.6. Throughput

To evaluate *throughput* for a fixed (s,t) pair, we implement the random-walk key-relaying scheme as a discrete-event simulation driven by a min-heap of timestamped events. To remain consistent with the two-phase protocol model, route discovery is treated as a preceding classical control phase that determines the realized payload route (with loop-erasure enabled, this route is LE(W)). The throughput metric $T_{s,t}$ counts only delivered payload fragment bits; scouting/control traffic is omitted from $T_{s,t}$ because it uses the classical channel and consumes no QKD-derived OTP key material. Each hop of a packet from node *u* to node *v* is split into: (i) *OTP key reservation* on link (u,v), which may incur waiting time computed from the link secret-key rate (SKR) and current key balance; (ii) a fixed-latency classical channel; and (iii) *receiver admission* into a finite relay buffer with FIFO backpressure. The simulator repeatedly pops the earliest event, advances the simulation clock, updates link/node state, and schedules successor events. Each undirected link e∈E generates QKD key material at a constant secret-key rate $g_{e}=1\;\mathrm{kbit}/s$ for all links. Key material is consumed in 256 bit units. Each hop spends 256 bit for one-time-pad (OTP) encryption on the traversed QKD link. We use a default inter-node classical latency of 5 ms. Simulations start with empty link buffers. We do not discard a warm-up period when calculating throughput; given a fixed simulation duration of 1000 s, its impact is unlikely to be large. We ignore classical network throughput (goodput) and protocol overhead, as QKD is the dominant bottleneck (e.g., 1 kbit/s vs. typical 1 Gbit/s classical links). Table 7 summarizes both the mean raw fragment throughput and the mean model-based extracted throughput across graphs and RW variants.

Raw fragment throughput varies only modestly across variants, but extracted throughput differs more because it inherits the pair-specific yield $\rho_{s,t}$. On NSFNET, mean raw throughput is about 2.0 kbit/s for all evaluated variants, whereas mean model-based extracted throughput ranges from roughly 0.62 to 0.64 kbit/s. On GÉANT, mean raw throughput ranges from about 1.70 to 1.77 kbit/s, while mean model-based extracted throughput rises from about 0.50 kbit/s for NB to about 0.56 kbit/s for HS. If the default design threshold τ=98% is used instead of pair-specific $\rho_{s,t}$, then $\rho^{\tau }=6/1024$ and the corresponding mean extracted-throughput proxy is only about 0.01 kbit/s. This mirrors the conservative-design efficiency collapse discussed after Table 9.

### 4.7. Comparison with Baseline

To put random flow on common footing with a topology-aware deterministic alternative, we introduce a node-disjoint multipath (MP) baseline. For each biconnected ordered pair (s,t), MP selects a set of *k* node-disjoint *s*–*t* paths $\left\{P_{1}^{s,t},\ldots ,P_{k}^{s,t}\right\}$ of minimum total length via Suurballe’s algorithm [27,28], which relies on repeated shortest-path computations (Dijkstra-class routines) over the known topology. Random-flow forwarding deliberately avoids such end-to-end shortest-path or disjoint-path computation at the source. The source XOR-splits each 256-bit final key *K* into *k* uniformly random 256-bit shares with $K=S_{1}⊕\ldots ⊕S_{k}$, transmits share $S_{i}$ along $P_{i}^{s,t}$ with each hop protected by a fresh QKD-derived 256-bit OTP key, and the destination reconstructs *K* as ${⨁}_{i=1}^{k}S_{i}$. The secrecy argument is the same as in Theorem 1, restricted to *k* shares across disjoint paths instead of *M* fragments along random walks. Under the same single-compromise threat model used for random flow, any compromised relay lies on at most one of the *k* disjoint paths and therefore observes at most one of the *k* shares. Since k−1 shares are needed to recover *K* in the information-theoretic sense, the worst-case MP fragment-exposure for any biconnected pair is exactly 1/k. Because MP is topology-aware, the source picks the largest feasible k=κ(s,t) per pair: with k=κ(s,t)≥2 on every biconnected pair, the worst-case fragment exposure under a single compromise is 1/κ(s,t)≤50% uniformly. Let $L_{i}^{s,t}=\left|P_{i}^{s,t}\right|$ denote the hop count of the *i*-th chosen disjoint path. One MP execution consumes $\sum_{i=1}^{k}L_{i}^{s,t}$ QKD link-key blocks of 256 bits and produces one 256-bit final key. The model-based efficiency, in the same units as $\eta_{s,t}$ (extracted output bits per consumed QKD-derived link-key bit), is therefore

$$\eta_{s,t}^{\mathrm{MP},k}:=\frac{1}{\sum_{i=1}^{k}L_{i}^{s,t}}.$$

This is the natural pairwise analogue of $\eta_{s,t}=\rho_{s,t}\;h_{s,t}^{-1}$ for the deterministic baseline: $\rho^{\mathrm{MP},k}\equiv 1/k$ (one final key per *k* transmitted shares) and the aggregate hop count is $\sum_{i}L_{i}^{s,t}=k\;{\bar{L}}^{s,t}$, so $\eta_{s,t}^{\mathrm{MP},k}=\left(1/k\right)\;\cdot \;{\left(k\;{\bar{L}}^{s,t}\right)}^{-1}=1/\;\sum_{i}L_{i}^{s,t}$. Recall from Section 4.3 that E(C)={(s,t):s,t∉C,sandtconnectedinG[V∖C]} is the set of ordered pairs that the cartel *C* does not disconnect, and that *C* is called *eligible* when E(C)≠∅. For an eligible cartel C⊆V of size *m*, define the per-scheme protected-pair sets

$$\begin{array}{cc} \Pi_{\mathrm{RF}}\left(C,\tau \right) & \;=\;\left\{\left(s,t\right)\in E\left(C\right)\;:\;p_{C}^{s,t}\leq \tau \right\},\\ \Pi_{\mathrm{MP}}\left(C,k\right) & \;=\;\left\{\left(s,t\right)\in E\left(C\right)\;:\;\exists \;i\in \left[k\right],\;P_{i}^{s,t}\cap C=\emptyset \right\}, \end{array}$$

and their normalized cardinalities (protected-pair coverage fractions)

$$\pi_{\mathrm{RF}}\left(C,\tau \right):=\frac{|\Pi_{\mathrm{RF}}\left(C,\tau \right)|}{\left|E(C)\right|},\;\pi_{\mathrm{MP}}\left(C,k\right):=\frac{|\Pi_{\mathrm{MP}}\left(C,k\right)|}{\left|E(C)\right|},$$

$$\pi_{\mathrm{RF}\setminus \mathrm{MP}}:=\frac{|\Pi_{\mathrm{RF}}\left(C,\tau \right)\setminus \Pi_{\mathrm{MP}}\left(C,k\right)|}{\left|E(C)\right|},\;\pi_{\mathrm{MP}\setminus \mathrm{RF}}:=\frac{|\Pi_{\mathrm{MP}}\left(C,k\right)\setminus \Pi_{\mathrm{RF}}\left(C,\tau \right)|}{\left|E(C)\right|}.$$

$\Pi_{\mathrm{RF}}$ is the complement of the affected set A(C,τ) inside E(C) at design threshold τ. $\Pi_{\mathrm{MP}}$ uses the path set fixed by Suurballe’s rule, so the deterministic baseline commits to its routing decision before the cartel is realized. We deliberately do not report a cartel-adaptive MP variant: identifying *C* at runtime falls outside both threat models considered here and outside the operational assumptions of the QKD-network layer, so the comparison of interest is between two schemes that the source can actually deploy. The two sets $\Pi_{\mathrm{RF}}$ and $\Pi_{\mathrm{MP}}$ are not nested in general. For a pair with κ(s,t)=1 and unique cut vertex *u*, the deterministic baseline collapses to single-path routing (k=1) through *u* and protects only against cartels disjoint from that one chosen path. Random flow still distributes fragments across many sampled walks that all traverse *u* but otherwise visit varying intermediate nodes; for cartels *C* with u∉C that intersect the deterministic shortest path but miss most RF-visited nodes, (s,t) can lie in $\Pi_{\mathrm{RF}}\setminus \Pi_{\mathrm{MP}}$. The same effect occurs for κ(s,t)≥2 pairs whose two deterministic disjoint paths are both intersected by a small cartel. When two short node-disjoint paths physically avoid *C*, MP protects (s,t) exactly. Random flow may still fail the τ-threshold on the same (s,t) if independent walks visit *C* with non-negligible probability, especially on graphs where most low-degree nodes lie outside the deterministic disjoint paths. Table 10 reports, for each topology and cartel size m∈{1,2,3}, the topology-wide means over eligible unordered cartels of $\pi_{\mathrm{RF}}$, $\pi_{\mathrm{MP}}$, $\pi_{\mathrm{RF}\setminus \mathrm{MP}}$, and $\pi_{\mathrm{MP}\setminus \mathrm{RF}}$. The left $\bar{\pi_{\mathrm{MP}}}$ column is τ-independent; the two right blocks give the RF-dependent metrics at the default τ=98% and at the stricter τ=95%. Table 9 contrasts the pair-mean efficiencies ${\bar{\eta }}^{\hat{\chi }}$ (HS with loop-erasure, M=1024) and $\bar{\eta^{\mathrm{MP},2}}$ over biconnected ordered pairs.

**Figure 8:**
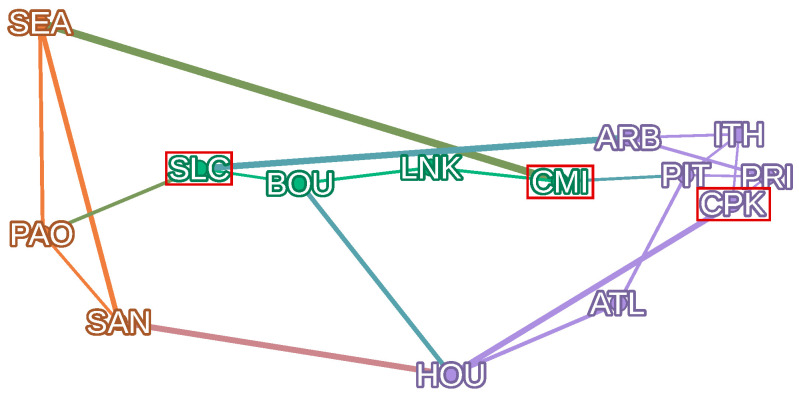
NSFNET with the m=3 cartel {CMI,SLC,CPK} highlighted. The highlighted node color marks the compromised cartel; other node colors are layout/community colors with no security meaning. At τ=98%, this cartel has $\pi_{\mathrm{RF}}=100.00\%$, $\pi_{\mathrm{MP}}=69.09\%$, and $\pi_{\mathrm{RF}\setminus \mathrm{MP}}=30.91\%$.

At the default τ=98%, random flow protects almost all eligible pairs and covers more pairs than the static Suurballe baseline in every reported row. The gap is largest on GÉANT, where $\bar{\pi_{\mathrm{RF}\setminus \mathrm{MP}}}$ reaches 9.42% at m=3, and smaller on NSFNET, where it reaches 4.44%. Conversely, $\bar{\pi_{\mathrm{MP}\setminus \mathrm{RF}}}$ is 0.00% in every τ=98% column. The τ=95% block shows that the two protected-pair sets are not nested in general: $\bar{\pi_{\mathrm{MP}\setminus \mathrm{RF}}}$ is small but nonzero, reaching at most 0.05% on NSFNET and 0.04% on GÉANT. That asymmetry is why we treat τ=98% as the default operating point in the remainder of the evaluation. Figure 8 illustrates a high-impact NSFNET 3-cartel, C={CMI,SLC,CPK}. Among all 143=364 unordered 3-cartels, this one has $\pi_{\mathrm{RF}\setminus \mathrm{MP}}=30.91\%$ (34 of 110 eligible ordered pairs) at both τ=98% and τ=95%, with $\pi_{\mathrm{RF}}=100.00\%$ and $\pi_{\mathrm{MP}}=69.09\%$. At τ=95%, it is the maximizing 3-cartel; at τ=98% the maximizing 3-cartel is {CMI,HOU,PRI}, with $\pi_{\mathrm{RF}\setminus \mathrm{MP}}=36.36\%$ (40 of 110 eligible ordered pairs). It is not a vertex cut, so the remaining NSFNET graph stays connected, but it hits many of the static Suurballe path sets chosen by MP. RF can still use surviving alternative walks, whereas static MP cannot change its preselected paths after the cartel is fixed.

At the default design threshold τ=98%, the conservative design yield is $\rho^{\tau }=g_{99.99\%}^{★}\left(1024,98\%\right)/1024=6/1024$, supporting θ=4 after the two-fragment privacy-amplification penalty. The corresponding topology-wide means are ${\bar{\eta }}^{\mathrm{RF},98}=0.24\%$ on NSFNET and 0.11% on GÉANT. For comparison, at τ=95% the corresponding ${\bar{\eta }}^{\mathrm{RF},95}$ values are 1.07% on NSFNET and 0.52% on GÉANT. Relative to the MP-2 column in Table 9, the τ=95% values are about 0.06× and 0.05×, respectively, whereas the τ=98% values are only about 0.01× on both topologies. The MP column in Table 9 is reported in extracted-key bits per link-key bit ($\theta_{\mathrm{MP}}=1$ per execution), while the RF column uses the safe-fragment yield ρ.

### 4.8. Scalability

**Figure 9:**
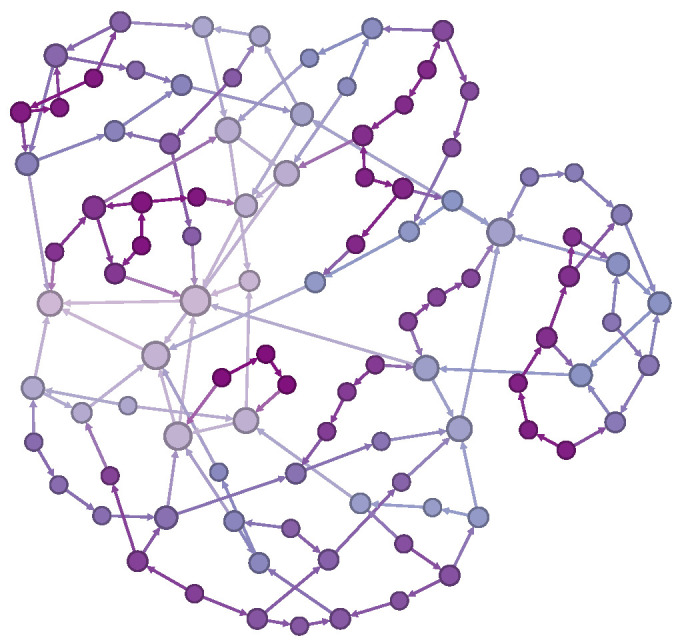
Synthetic graph with 99 nodes. Darker nodes have higher sequence numbers, and edge directions indicate insertion order.

To study scaling trends beyond the two deployed-analog topologies above, we generate a synthetic 2-vertex-connected graph with 99 nodes and 143 edges by placing clusters of 3 nodes at randomly chosen nearby coordinates and connecting each cluster to 2–3 of its 6 closest neighbors; generation is retried if the graph is not 2-vertex-connected. In Figure 9, darker nodes have higher sequence numbers (they appear later in snapshots), and edge directions indicate the insertion step when the edge was added. The graph has an average degree of ≈2.9, comparable to NSFNET and GÉANT. Furthermore, we bias edge creation toward geographically closer nodes. Each node is also assigned a sequence number so we can take snapshots at n=3,6,…,99 such that the snapshots maintain the same properties.

Table 11 summarizes basic structural metrics of the synthetic graph.

**Figure 10:**
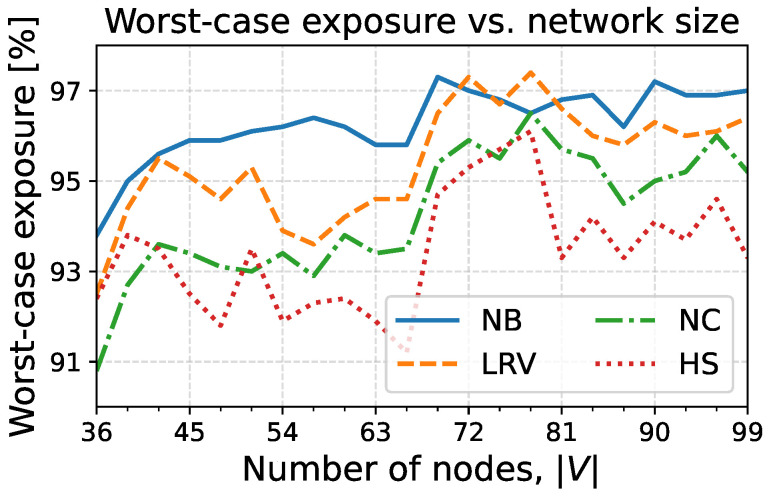
Worst-case exposure on synthetic graph snapshots as the network size grows.

Table 12 reports single-relay exposure on the final 99-node snapshot and the peak worst-case exposure over all synthetic snapshots. To evaluate whether worst-case exposure $\max_{s,t}\chi_{s,t}$ grows with network size, we measured it on successive snapshots and report the results in Figure 10. Although the worst-case exposure remains very high throughout, we do not observe a clear monotonic increase with |V|. Moreover, for the non-backtracking variants, the peak maximum is not attained at the final 99-node snapshot: NB peaks at 97.3% on the 69-node snapshot, while LRV, NC, and HS peak at 97.4%, 96.5%, and 96.1%, respectively, on the 78-node snapshot. This suggests that, at least for our generated graph family, worst-case exposure is driven more by structural bottlenecks than by network size alone.

**Figure 11:**
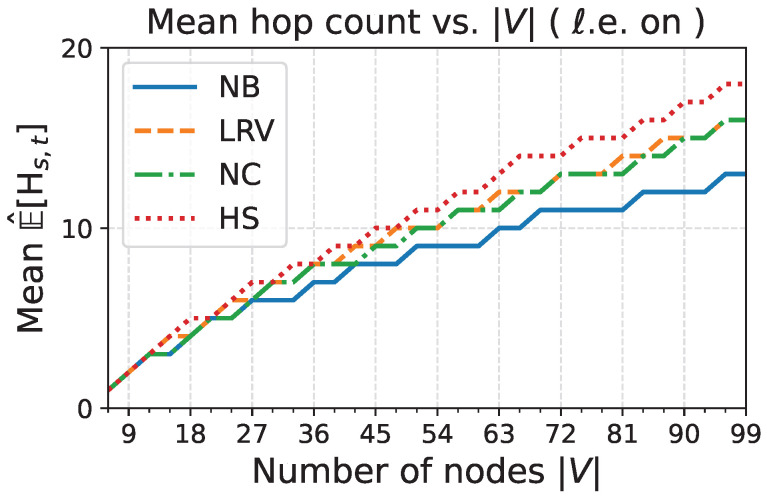
Average expected hop count by graph and RW variant with loop-erasure.

As shown in Figure 11, the average loop-erased expected hop count over all (s,t) pairs grows approximately linearly with network size on the synthetic snapshots. Table 13 gives hop-count statistics on the final 99-node snapshot. On that graph, the average shortest-path length over 9702 ordered pairs is 4.8 (Table 11), whereas the average mean hop count is 10–18 with loop-erasure and 41–128 without it. The average length of the shortest path when original shortest-path nodes are removed is 7.5, still significantly below 10–18 post-loop-erasure. We include this metric as a proxy for the length of a second path in a node-disjoint routing scenario; it is an upper bound on the second-path length produced by Suurballe’s minimum-total-length disjoint-pair algorithm [27,28], since sequential shortest-path-then-remove need not coincide with the jointly optimal pair. Even this alternate-path baseline is shorter than the loop-erased random-flow routes on the generated graph.

Table 14 and Table 15 extend the efficiency and throughput analysis to the final synthetic snapshot. On the generated graph, mean raw throughput stays nearly flat across variants, at about 1.97–2.01 kbit/s, but mean model-based extracted throughput increases from about 0.41 kbit/s for NB to about 0.49 kbit/s for HS.

### 4.9. Evaluation Summary

The numerical results above support five main conclusions. First, HS with loop-erasure is the only evaluated random-flow variant that we would carry forward as a protocol candidate. It gives the lowest worst-case exposure among the evaluated variants, while NC is only a diagnostic comparison because it assumes global node-set knowledge. R, NB, and LRV are therefore best read as baselines rather than implementer-facing recommendations. Second, loop-erasure is what makes the random-flow route lengths plausible. On GÉANT, the mean HS route length falls from 19.8 hops to 8.7 hops after loop-erasure; on the synthetic graph, it falls from 49 hops to 18 hops. The same pattern holds for the other nontrivial random-walk variants. Nevertheless, these loop-erased routes remain longer than shortest-path and alternate-path proxies for topology-aware node-disjoint routing [27,28], so random flow should not be interpreted as a route-efficiency improvement over a controller that already knows suitable disjoint paths. Third, the efficiency and throughput results separate pair-specific behavior from conservative worst-case parameterization. With pair-specific exposure estimates, the model-based extracted-throughput proxy is tightly clustered on NSFNET, at 0.62–0.64 kbit/s, while on GÉANT it increases from 0.50 kbit/s for NB to 0.56 kbit/s for HS. With the default τ=98% design threshold, however, the conservative RF efficiency falls to 0.24% on NSFNET and 0.11% on GÉANT, about 0.01× the MP-2 baseline in both cases. The method is therefore attractive only when the deployment accepts this worst-case cost, uses pair-specific parameterization, or treats random flow as a topology-oblivious fallback rather than as the efficiency optimum. Fourth, the comparison with static multipath is mixed rather than one-sided. Static Suurballe-style multipath remains the natural benchmark when route computation and topology knowledge are acceptable. At the same time, at τ=98%, random flow protects almost all eligible pairs and covers more pairs than the static multipath baseline in every reported protected-pair row; on GÉANT with m=3, the mean RF-only coverage reaches 9.42% and the mean MP-only coverage is 0.00%. At τ=95%, the protected-pair sets are not nested, so neither method dominates the other for every cartel placement. Fifth, the two- and three-relay GÉANT cartel analysis is a sensitivity study, not a full multi-relay ITS guarantee. For arbitrary eligible cartels, the median affected share is often zero at the higher design thresholds, but the maximum column shows that favorable cartel placements can still make the single-relay design exposure too optimistic: at τ=98%, the worst three-node cartel affects 19.49% of eligible ordered pairs, whereas arbitrary three-node placements affect only 0.02% on average. When this happens, the remaining claim is the computational fallback of Section 3.5, not the information-theoretic extraction claim.

## 5. Conclusions

This paper presents random flow as a topology-oblivious QKD key-relaying design that trades extracted-key efficiency for local forwarding, simpler operation, and reduced dependence on precomputed disjoint paths. Random flow fragments key material, sends fragments along independently sampled random walks, and applies seeded privacy amplification at the destination. The evaluation identifies HS forwarding with scouting-based loop erasure as the useful protocol candidate. On GÉANT, HS reduces worst-case single-relay exposure to 91.3%, compared with 98.3% for simple random walk, and loop erasure reduces mean HS route length from 19.8 to 8.7 hops. The main observation is that a simple local construction can exploit partial disjointness: although static node-disjoint multipath remains the natural benchmark when topology knowledge and route computation are acceptable, random flow achieves competitive protected-pair coverage under the evaluated cartel metric. The price is efficiency under conservative worst-case parameterization. At τ=98%, RF efficiency is low compared with MP-2, but the throughput simulations suggest that this overhead is partly mitigated in the two-KME communication scenario because random walks spread fragment load over the network rather than concentrating it on a fixed route set. The computational fallback in Section 3.5 further explains why a large *M* remains useful even when the ITS extracted block is small. Random flow therefore points to a different design target for Q-KMS systems: not maximum route efficiency, but simple, decentralized, auditable key relaying with minimal routing state. This is particularly relevant in deployments where global topology knowledge, centralized orchestration, or per-pair path computation are operational liabilities. Several extensions could improve scalability without abandoning this design direction. Signed forwarding histories would make realized-path accounting auditable. Scouting can be extended so that the source samples several candidate walks and relays payload only over a selected subset, for example the most mutually disjoint scouts. Larger networks could also be handled hierarchically by dividing the topology into modularity communities and applying random flow within or between those regions.

## Figures and Tables

**Table 1:** Maximum *g* values such that P[#≥g]>α holds for various exposure values χ. These values determine the guaranteed safe-fragment count used by the ITS parameter choice.

	$g_{99\%}^{★}\left(M,\chi \right)$						$g_{99.99\%}^{★}\left(M,\chi \right)$					
M	98%	95%	90%	85%	80%	75%	98%	95%	90%	85%	80%	75%
32	0	0	0	1	2	3	0	0	0	0	0	0
64	0	0	2	4	6	8	0	0	0	1	3	5
128	0	1	6	10	16	21	0	0	2	6	10	15
256	1	5	15	26	37	48	0	2	10	19	29	39
512	4	15	36	59	82	106	1	9	28	48	70	93
1024	11	36	81	128	175	224	6	27	69	113	159	206

**Table 2:** Topology overview. ASP is the average shortest-path length. The final columns report the percentage of ordered endpoint pairs with vertex connectivity κ(s,t) at least 2 or 3.

Graph	Nodes	Edges	Diam.	Avg. Deg.	ASP	κ≥2	κ≥3
NSFNET	14	21	3	3.00	2.143	100%	72.5%
GÉANT	43	59	12	2.74	4.682	70.1%	5.0%

**Table 3:** RW variant $\max_{s,t}\hat{\chi }$ values (in %) on GÉANT and corresponding intermediate vertex *v* under the single-relay metric.

Variant	Max $\hat{\chi }$	*s*	*t*	*v*	Avg $\hat{\chi }$	Median $\hat{\chi }$
R	98.3	COR	BIL	PAR	71.0	78.1
NB	95.7	BIL	COR	PAR	66.9	72.7
LRV	95.3	POR	COR	PAR	67.0	73.7
NC	93.6	POR	COR	PAR	65.8	72.8
HS	91.3	MAD	COR	PAR	65.0	71.7

**Table 4:** Exposure overview by graph and RW variant under the single-relay metric. For each graph and variant, we report the worst-case pair exposure $\max_{s,t}\hat{\chi }$ and the median $\hat{\chi }$ over all ordered source–target pairs.

Graph	Max Exposure $\hat{\chi }$ [%]					Median Exposure $\hat{\chi }$ [%]				
	R	NB	LRV	NC	HS	R	NB	LRV	NC	HS
NSFNET	81.2	78.1	77.4	72.8	71.0	52.6	50.9	52.8	50.8	51.6
GÉANT	98.3	95.7	95.3	93.6	91.3	78.1	72.7	73.7	72.8	71.7

**Table 5:** Two- and three-relay compromise analysis on GÉANT using HS. For each design threshold τ, the table reports the fraction of eligible pairs that are *affected* (|A(C,τ)|/|E(C)|), over all cartels *C*.

τ	Two-Node Cartel			Three-Node Cartels		
	Median	Average	Maximum	Median	Average	Maximum
75%	0.61%	5.56%	60.98%	7.31%	13.24%	68.42%
80%	0.00%	3.17%	54.94%	2.88%	8.76%	65.65%
85%	0.00%	1.44%	41.83%	0.47%	4.92%	60.93%
90%	0.00%	0.41%	31.65%	0.00%	2.00%	53.17%
95%	0.00%	0.03%	5.79%	0.00%	0.34%	33.47%
97%	0.00%	0.00%	0.79%	0.00%	0.07%	24.62%
98%	0.00%	0.00%	0.00%	0.00%	0.02%	19.49%
99%	0.00%	0.00%	0.00%	0.00%	0.00%	3.33%

**Table 6:** Topology-wide mean RW efficiency by graph and variant, in percent. Each row reports ${\bar{\eta }}^{\tau }$ (fixed τ from Table 4 and $\rho^{\tau }=g_{\alpha }^{★}\left(M,\tau \right)/M$) or ${\bar{\eta }}^{\hat{\chi }}$ (pairwise measured ${\hat{\chi }}_{s,t}^{\left(m\right)}$). The left block uses hop counts from the original walk, while the right block uses hop counts after loop-erasure. In both blocks, exposure is measured on the original walk and M=1024.

Graph	Mean	Loop-Erasure *off* [%]				Loop-Erasure *on* [%]			
		NB	LRV	NC	HS	NB	LRV	NC	HS
NSFNET	${\bar{\eta }}^{\tau }$	6.49	7.07	8.69	9.47	7.25	7.23	9.12	9.63
	${\bar{\eta }}^{\hat{\chi }}$	26.5	26.8	26.9	27.1	27.6	27.1	27.4	27.2
GÉANT	${\bar{\eta }}^{\tau }$	0.27	0.36	0.54	0.79	0.48	0.49	0.76	1.09
	${\bar{\eta }}^{\hat{\chi }}$	9.7	10.2	10.2	10.2	12.1	11.6	11.8	11.7

**Table 7:** Mean throughput by graph and RW variant. The top block reports raw fragment throughput $T_{s,t}$ in kbit/s. The bottom block reports mean model-based extracted throughput $R_{s,t}=T_{s,t}\rho_{s,t}$ in kbit/s under the simplified entropy model.

Metric	Graph	NB	LRV	NC	HS
$T_{s,t}$	NSFNET	2.03	2.02	2.05	2.03
	GÉANT	1.70	1.70	1.72	1.77
$R_{s,t}$	NSFNET	0.64	0.62	0.64	0.64
	GÉANT	0.50	0.50	0.51	0.56

**Table 8:** Estimated expected hop count by graph and RW variant. Let $H_{s,t}$ be the hop count in the sampled walk from *s* to *t*. We sample W=1000 walks per biconnected ordered source–target pair and variant and report the average of mean, median, and maximum of $H_{s,t}$ over all biconnected (s,t) pairs, distinguishing whether loop-erasure is toggled on.

Graph	Metric	Without Loop-Erasure					With Loop-Erasure				
		R	NB	LRV	NC	HS	R	NB	LRV	NC	HS
NSFNET	Mean	6.4	4.5	4.0	4.1	3.9	3.1	3.4	3.7	3.6	3.7
	Median	5	4	3	4	3	3	3	3	3	3
	Max	40	23	9	12	10	7	8	8	8	8
GÉANT	Mean	64.4	32.2	18.4	18.9	19.8	6.3	7.2	8.3	8.1	8.7
	Median	42	22	15	16	15	6	6	7	7	8
	Max	512	243	70	83	130	18	21	23	23	24

**Table 9:** Pair-mean efficiency comparison. RF column reproduces ${\bar{\eta }}^{\hat{\chi }}$ from Table 6 (HS, with loop-erasure, M=1024). MP column reports $\bar{\eta^{\mathrm{MP},2}}=\bar{1/(L_{1}+L_{2})}$ averaged over biconnected ordered pairs using Suurballe shortest disjoint-pair paths.

Graph	${\bar{\eta }}_{\mathrm{HS}}^{\hat{\chi }}$ [%]	$\bar{\eta^{\mathrm{MP},2}}$ [%]	RF/MP
NSFNET	27.3	18.1	1.5
GÉANT	11.8	11.1	1.1

**Table 10:** Protected-pair coverage of random flow (HS with loop-erasure, M=1024) and static Suurballe k=κ(s,t) multipath. Each cell is a mean over eligible unordered cartels *C* of size *m*. The $\bar{\pi_{\mathrm{MP}}}$ column is independent of the RF design threshold τ; the two right blocks report RF-dependent metrics at τ=98% (default) and τ=95%.

Graph	*m*	$\bar{\pi_{\mathrm{MP}}}$	τ=98%			τ=95%		
			$\bar{\pi_{\mathrm{RF}}}$	$\bar{\pi_{\mathrm{RF}\setminus \mathrm{MP}}}$	$\bar{\pi_{\mathrm{MP}\setminus \mathrm{RF}}}$	$\bar{\pi_{\mathrm{RF}}}$	$\bar{\pi_{\mathrm{RF}\setminus \mathrm{MP}}}$	$\bar{\pi_{\mathrm{MP}\setminus \mathrm{RF}}}$
NSFNET	1	100.00%	100.00%	0.00%	0.00%	100.00%	0.00%	0.00%
NSFNET	2	98.94%	100.00%	1.06%	0.00%	100.00%	1.06%	0.00%
NSFNET	3	95.55%	100.00%	4.44%	0.00%	99.62%	4.12%	0.05%
GÉANT	1	97.56%	100.00%	2.44%	0.00%	100.00%	2.44%	0.00%
GÉANT	2	94.18%	100.00%	5.82%	0.00%	99.96%	5.79%	0.01%
GÉANT	3	90.56%	99.98%	9.42%	0.00%	99.65%	9.13%	0.04%

**Table 11:** Topology overview of the synthetic graph. ASP is the average shortest-path length. The final columns report the percentage of ordered endpoint pairs with vertex connectivity κ(s,t) at least 2 or 3.

Graph	Nodes	Edges	Diam.	Avg. Deg.	ASP	κ≥2	κ≥3
Generated	99	143	10	2.89	4.784	100%	21.1%

**Table 12:** Single-relay exposure on the synthetic graph (single-relay metric). For each variant, we report the worst-case pair exposure $\max_{s,t}\hat{\chi }$ at the final 99-node snapshot, the peak $\max_{s,t}\hat{\chi }$ over all snapshots, the snapshot size at that peak, and the median $\hat{\chi }$ over biconnected (s,t) pairs at n=99.

Variant	Max $\hat{\chi }$ at n=99 [%]	Peak Max $\hat{\chi }$ [%]	Peak *n*	Median $\hat{\chi }$ at n=99 [%]
R	98.5	98.5	99	84.8
NB	97.0	97.3	69	78.5
LRV	96.4	97.4	78	77.2
NC	95.2	96.5	78	76.7
HS	93.3	96.1	78	72.8

**Table 13:** Estimated expected hop count on the final 99-node synthetic graph. Let $H_{s,t}$ be the hop count in the sampled walk from *s* to *t*. We report the average of mean, median, and maximum of $H_{s,t}$ over all biconnected (s,t) pairs, and distinguish whether loop-erasure is toggled on.

Graph	Metric	Without Loop-Erasure					With Loop-Erasure				
		R	NB	LRV	NC	HS	R	NB	LRV	NC	HS
Generated	Mean	128	68	41	42	49	10	13	16	16	18
	Median	85	46	34	35	36	9	11	14	14	16
	Max	1027	516	163	181	357	33	42	54	53	58

**Table 14:** Topology-wide mean RW efficiency on the final 99-node synthetic graph, in percent. Rows report ${\bar{\eta }}^{\tau }$ or ${\bar{\eta }}^{\hat{\chi }}$ as in Table 6. The left block uses hop counts from the original walk, while the right block uses hop counts after loop-erasure. In both blocks, exposure is measured on the original walk and M=1024.

Graph	Mean	Loop-Erasure *off* [%]				Loop-Erasure *on* [%]			
		NB	LRV	NC	HS	NB	LRV	NC	HS
Generated	${\bar{\eta }}^{\tau }$	0.05	0.06	0.09	0.10	0.12	0.09	0.15	0.16
	${\bar{\eta }}^{\hat{\chi }}$	3.5	3.7	3.7	3.7	4.6	4.4	4.4	4.4

**Table 15:** Mean throughput on the final 99-node synthetic graph. The top block reports raw fragment throughput $T_{s,t}$ in kbit/s. The bottom block reports mean model-based extracted throughput $R_{s,t}=T_{s,t}\rho_{s,t}$ in kbit/s under the simplified entropy model.

Metric	Graph	NB	LRV	NC	HS
$T_{s,t}$	Generated	1.97	1.97	1.97	2.01
$R_{s,t}$	Generated	0.41	0.43	0.44	0.49

## Data Availability

The graph edge lists and simulation source code supporting the findings of this study are publicly available at https://github.com/LUMII-Syslab/random-walk-key-relaying (accessed on 4 February 2026).

## References

[1] Shannon C.E. (1949). Communication Theory of Secrecy Systems. Bell Syst. Tech. J..

[2] Bennett C.H., Brassard G. (2014). Quantum Cryptography: Public Key Distribution and Coin Tossing. Theor. Comput. Sci..

[3] Shor P.W., Preskill J. (2000). Simple Proof of Security of the BB84 Quantum Key Distribution Protocol. Phys. Rev. Lett..

[4] Peev M., Pacher C., Alléaume R., Barreiro C., Bouda J., Boxleitner W., Debuisschert T., Diamanti E., Dianati M., Dynes J.F. (2009). The SECOQC Quantum Key Distribution Network in Vienna. New J. Phys..

[5] Stucki D., Legré M., Buntschu F., Clausen B., Felber N., Gisin N., Henzen L., Junod P., Litzistorf G., Monbaron P. (2011). Long-Term Performance of the SwissQuantum Quantum Key Distribution Network in a Field Environment. New J. Phys..

[6] Chen T.Y., Jiang X., Tang S.B., Zhou L., Yuan X., Zhou H., Wang J., Liu Y., Chen L.K., Liu W.Y. (2021). Implementation of a 46-Node Quantum Metropolitan Area Network. npj Quantum Inf..

[7] Gisin N., Ribordy G., Tittel W., Zbinden H. (2002). Quantum Cryptography. Rev. Mod. Phys..

[8] Huang D., Huang P., Lin D., Zeng G. (2016). Long-Distance Continuous-Variable Quantum Key Distribution by Controlling Excess Noise. Sci. Rep..

[9] Pittaluga M., Lo Y.S., Brzosko A., Woodward R.I., Scalcon D., Winnel M.S., Roger T., Dynes J.F., Owen K.A., Juárez S. (2025). Long-Distance Coherent Quantum Communications in Deployed Telecom Networks. Nature.

[10] Salvail L., Peev M., Diamanti E., Alléaume R., Lütkenhaus N., Länger T. (2010). Security of Trusted Repeater Quantum Key Distribution Networks. J. Comput. Secur..

[11] Dervisevic E., Tankovic A., Fazel E., Kompella R., Fazio P., Voznak M., Mehic M. (2025). Quantum Key Distribution Networks-Key Management: A Survey. ACM Comput. Surv..

[12] Cvitić I., Peraković D., Nolasco A. (2025). Overview of Routing Approaches in Quantum Key Distribution Networks. arXiv.

[13] Moy J. (1998). RFC 2328: OSPF Version 2. https://www.rfc-editor.org/info/rfc2328/.

[14] Drif Y., Bedhief I., Chatzinotas S. (2025). Distributed Key Relay: OSPF for Effective QKD. IEEE Commun. Stand. Mag..

[15] Liao S.K., Cai W.Q., Liu W.Y., Zhang L., Li Y., Ren J.G., Yin J., Shen Q., Cao Y., Li Z.P. (2017). Satellite-to-Ground Quantum Key Distribution. Nature.

[16] Pirandola S., Laurenza R., Ottaviani C., Banchi L. (2017). Fundamental Limits of Repeaterless Quantum Communications. Nat. Commun..

[17] Lucamarini M., Yuan Z.L., Dynes J.F., Shields A.J. (2018). Overcoming the Rate–Distance Limit of Quantum Key Distribution without Quantum Repeaters. Nature.

[18] Minder M., Pittaluga M., Roberts G.L., Lucamarini M., Dynes J.F., Yuan Z.L., Shields A.J. (2019). Experimental Quantum Key Distribution beyond the Repeaterless Secret Key Capacity. Nat. Photonics.

[19] Elliott C. (2002). Building the Quantum Network. New J. Phys..

[20] (2019). Quantum Key Distribution (QKD); Protocol and Data Format of REST-Based Key Delivery API.

[21] Rescorla E. (2018). RFC 8446: The Transport Layer Security (TLS) Protocol Version 1.3. https://www.rfc-editor.org/info/rfc8446/.

[22] Kaufman C., Hoffman P., Nir Y., Eronen P., Kivinen T. (2014). RFC 7296: Internet Key Exchange Protocol Version 2 (IKEv2). https://www.rfc-editor.org/info/rfc7296/.

[23] Dowling B., Hansen T.B., Paterson K.G., Ding J., Tillich J.P. (2020). Many a Mickle Makes a Muckle: A Framework for Provably Quantum-Secure Hybrid Key Exchange. Post-Quantum Cryptography.

[24] Beals T.R., Sanders B.C. (2008). Distributed Relay Protocol for Probabilistic Information-Theoretic Security in a Randomly-Compromised Network. Proceedings of the International Conference on Information Theoretic Security.

[25] Wen H., Han Z., Zhao Y., Guo G., Hong P. (2009). Multiple Stochastic Paths Scheme on Partially-Trusted Relay Quantum Key Distribution Network. Sci. China Ser. Inf. Sci..

[26] Kiktenko E.O., Tayduganov A., Fedorov A.K. (2024). Routing Algorithm Within the Multiple Non-Overlapping Paths’ Approach for Quantum Key Distribution Networks. Entropy.

[27] Suurballe J.W. (1974). Disjoint Paths in a Network. Networks.

[28] Bhandari R. (1998). Survivable Networks: Algorithms for Diverse Routing.

[29] Kumar P., Kundu N.K., Kar B. (2024). Quantum Key Distribution Routing Protocol in Quantum Networks: Overview and Challenges. arXiv.

[30] Yao J., Wang Y., Li Q., Mao H., El-Latif A.A.A., Chen N. (2022). An Efficient Routing Protocol for Quantum Key Distribution Networks. Entropy.

[31] Álvarez Roa M., Stan C., Verschoor S., Tafur Monroy I., Rommel S. (2025). Decentralized Key Distribution versus On-Demand Relaying for QKD Networks. J. Opt. Commun. Netw..

[32] Stan C., Verchere D., Olmos J.J.V., Tafur Monroy I., Rommel S. (2025). Dynamic-Threshold-Based Pre-Relaying for Enhanced Key Allocation in Quantum-Secured Networks. J. Opt. Commun. Netw..

[33] Wang M., Li J., Xue K., Li R., Yu N., Li Y., Liu Y., Sun Q., Lu J. (2023). A Segment-Based Multipath Distribution Method in Partially-Trusted Relay Quantum Networks. IEEE Commun. Mag..

[34] Le Q.C., Bellot P., Demaille A., Chen L., Mu Y., Susilo W. (2008). Towards the World-Wide Quantum Network. Information Security Practice and Experience.

[35] Le Quoc C., Bellot P., Demaille A. (2007). Stochastic Routing in Large Grid-Shaped Quantum Networks. Proceedings of the 2007 IEEE International Conference on Research, Innovation and Vision for the Future.

[36] Ghourab E.M., Azab M., Gračanin D. (2025). QKD Routing Scheme for a Zero-Trust Network System: A Moving Target Defense Approach. Big Data Cogn. Comput..

[37] Braunstein S.L., Pirandola S. (2012). Side-Channel-Free Quantum Key Distribution. Phys. Rev. Lett..

[38] Lo H.K., Curty M., Qi B. (2012). Measurement-Device-Independent Quantum Key Distribution. Phys. Rev. Lett..

[39] Liu Y., Chen T.Y., Wang L.J., Liang H., Shentu G.L., Wang J., Cui K., Yin H.L., Liu N.L., Li L. (2013). Experimental Measurement-Device-Independent Quantum Key Distribution. Phys. Rev. Lett..

[40] Fan-Yuan G.J., Lu F.Y., Wang S., Yin Z.Q., He D.Y., Chen W., Zhou Z., Wang Z.H., Teng J., Guo G.C. (2022). Robust and Adaptable Quantum Key Distribution Network without Trusted Nodes. Optica.

[41] Yan W., Zheng X., Wen W., Lu L., Du Y., Lu Y.Q., Zhu S., Ma X.S. (2025). A Measurement-Device-Independent Quantum Key Distribution Network Using Optical Frequency Comb. npj Quantum Inf..

[42] Cao Y., Zhao Y., Li J., Lin R., Zhang J., Chen J. (2021). Hybrid Trusted/Untrusted Relay-Based Quantum Key Distribution Over Optical Backbone Networks. IEEE J. Sel. Areas Commun..

[43] Yu X., Liu Y., Zou X., Cao Y., Zhao Y., Nag A., Zhang J. (2022). Secret-Key Provisioning With Collaborative Routing in Partially-Trusted-Relay-based Quantum-Key-Distribution-Secured Optical Networks. J. Light. Technol..

[44] Rass S., Mehic M., Voznak M., König S. (2024). Hacking the Least Trusted Node: Indirect Eavesdropping in Quantum Networks. IEEE Access.

[45] Lydersen L., Wiechers C., Wittmann C., Elser D., Skaar J., Makarov V. (2010). Hacking Commercial Quantum Cryptography Systems by Tailored Bright Illumination. Nat. Photonics.

[46] Ford L.R., Fulkerson D.R. (1962). Flows in Networks.

[47] Alon N., Benjamini I., Lubetzky E., Sodin S. (2007). Non-Backtracking Random Walks Mix Faster. Commun. Contemp. Math..

[48] Akash A.K., Fekete S., Lee S.K., López-Ortiz A., Maftuleac D., McLurkin J. (2017). Lower Bounds for Graph Exploration Using Local Policies. J. Graph Algorithms Appl..

[49] Grover L.K. (1996). A Fast Quantum Mechanical Algorithm for Database Search. Proceedings of the Twenty-Eighth Annual ACM Symposium on Theory of Computing-STOC ’96.

[50] Fung C.H.F., Ma X., Chau H.F. (2010). Practical Issues in Quantum-Key-Distribution Postprocessing. Phys. Rev. A—At. Mol. Opt. Phys..

[51] Bennett C.H., Brassard G., Robert J.M. (1988). Privacy Amplification by Public Discussion. SIAM J. Comput..

[52] Renner R., König R., Kilian J. (2005). Universally Composable Privacy Amplification Against Quantum Adversaries. Theory of Cryptography.

[53] Tomamichel M., Schaffner C., Smith A., Renner R. (2011). Leftover Hashing Against Quantum Side Information. IEEE Trans. Inf. Theory.

[54] Stinson D.R., Paterson M.B. (2005). Cryptography: Theory and Practice.

[55] Lawler G.F., Limic V. (2010). Random Walk: A Modern Introduction.

[56] Wilson D.B. (1996). Generating Random Spanning Trees More Quickly than the Cover Time. Proceedings of the Twenty-Eighth Annual ACM Symposium on Theory of Computing-STOC ’96.

[57] Mehic M., Niemiec M., Rass S., Ma J., Peev M., Aguado A., Martin V., Schauer S., Poppe A., Pacher C. (2020). Quantum Key Distribution: A Networking Perspective. ACM Comput. Surv. (CSUR).

[58] Mukherjee B., Ramamurthy S., Banerjee D., Mukherjee A. (2002). Some Principles for Designing a Wide-Area Optical Network. Proceedings of the INFOCOM ’94 Conference on Computer Communications.

[59] GÉANT (2023). GN4-3N. Web Page Describing the GN4-3N Project (2019–2023); Topology Diagram Consulted to Reconstruct Node/Edge Lists (Links > 1000 km Removed in Our Processed Graph). https://network.geant.org/gn4-3n/.

[60] Xuereb A. (2023). Towards an Ultra-Secure Communication Network for the EU. GÉANT CONNECT. Online Article Discussing EuroQCI-Oriented Ultra-Secure Networking Efforts Including QKD. https://connect.geant.org/2023/12/13/towards-an-ultra-secure-communication-network-for-the-eu.

